# RF-Sputtered β-Ga_2_O_3_ Thin Films for Solar-Blind UV Detection: Progress, Challenges, and Future Perspectives

**DOI:** 10.3390/ma19102165

**Published:** 2026-05-21

**Authors:** Pramod Mandal, Shagolsem Romeo Meitei, Anand Pandey

**Affiliations:** 1 Department of Graphic Arts and Photophysics, Faculty of Chemical Technology, University of Pardubice, 532 10 Pardubice, Czech Republic; pramodkumar.mandal@upce.cz; 2 Electronics and Communication Engineering Department, National Institute of Technology Andhra Pradesh, Tadepalligudem 534101, India; romeo.shagolsem@nitandhra.ac.in; 3 Department of Physics, Chemistry and Biology (IFM), Linköping University, 581 83 Linköping, Sweden

**Keywords:** *β*-Ga_2_O_3_, PVD RF magnetron sputtering, solar-blind UV photodetector (SB-UVPDs), optoelectronics, wide bandgap semiconductor

## Abstract

This review presents a comprehensive and thorough evaluation of recent developments in physical vapour deposition (PVD) radiofrequency (RF)-sputtered *β*-Ga_2_O_3_ thin-film-based solar-blind ultraviolet (UV) photodetectors (SB-UVPDs), emphasizing their potential for next-generation optoelectronic applications. The review highlights different photodetector architectures, the performance characteristics of SB-UVPDs, and an overview of the attributes of *β*-Ga_2_O_3_ that make it a promising wide-bandgap semiconductor for next-generation devices. Additionally, the working principle of the PVD RF magnetron sputtering technique is discussed briefly, with a particular focus on the influence of deposition parameters, including sputtering power, gas pressure, deposition time, target-to-substrate distance, and substrate temperature, on the resulting film’s crystallinity and morphology and the optical quality of SB-UVPDs. Moreover, the impact of post-deposition treatments, such as post-annealing and elemental doping, is also discussed here for SB-UVPDs. And finally, the electrical performance characteristics of SB-UVPDs are discussed categorically based on deposition parameters. Overall, this review establishes that PVD RF magnetron sputtering is a highly versatile and controllable technique for fabricating high-quality *β*-Ga_2_O_3_ thin film-based SB-UVPDs. By carefully optimizing deposition and post-processing parameters, the optoelectronic performance of *β*-Ga_2_O_3_-based SB-UVPDs can be effectively tuned, enabling their integration into next-generation high-performance optoelectronic and photonic systems.

## 1. Introduction

**Figure 1:**
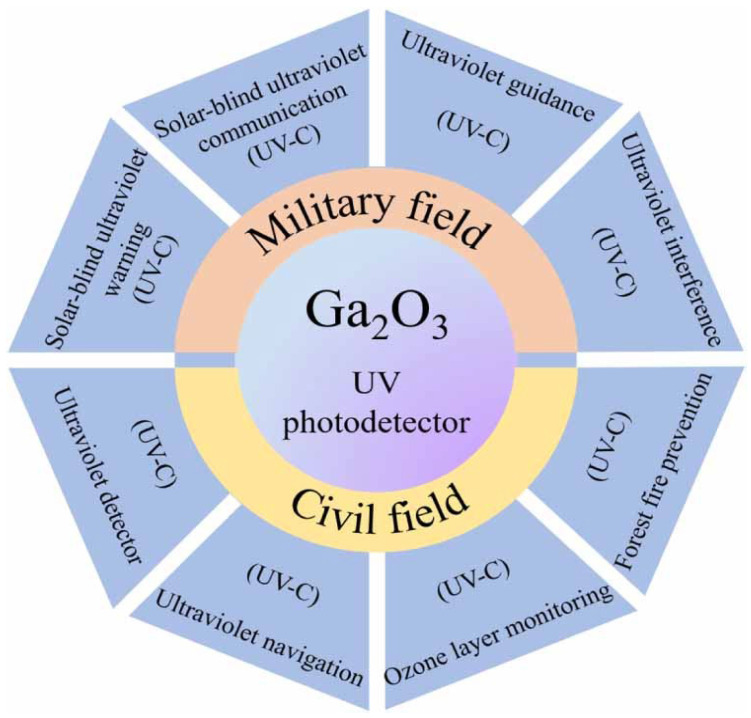
Ga_2_O_3_-based UV photodetector applications for military and civil fields. Adapted with permission from reference [16]. Copyright © 2024. IOP Publishing.

The development of ultra-wide bandgap semiconductors (WBGs) has received significant attention for applications in ultraviolet (UV) nanoscale electronics and optoelectronic devices [1,2,3]. Recently, among various WBGs, *β*-gallium oxide (Ga_2_O_3_) has been extensively used for solar-blind UV photodetector (SB-UVPDs) applications in missile tracking, Corona detection, flame detection in gas stations, oil spill detection of tankers at sea, the detection of volcanic eruptions, UV astronomy, ozone layer shrinking, etc., and is considered for next-generation device applications [4,5,6,7]. It is well known that more than 99% of the solar energy spectrum lies between 150 and 4000 nm, and approximately 7% of this band falls within the UV spectral range [8]. UV spectral regions are usually defined as the wavelength range 10–400 nm. UV spectral areas are commonly referred to as UV-A (320–400 nm), UV-B (280–320 nm), UV-C (100–280 nm), and severe UV (10–120 nm) wavelengths [9,10]. The UV spectral window spanning wavelengths from 200 to 280 nm is termed the “solar-blind region” [5,11,12]. At the Earth’s surface, the solar-blind region is characterized by an extremely low background signal because atmospheric constituents effectively absorb most of the incoming solar radiation in this wavelength range [13]. As a result, UV emissions in the solar-blind region can be detected with minimal interference from sunlight. SB-UVPDs operating in this band significantly suppress false signal generation by enabling the detection of weak UV emissions against a strong terrestrial background [14,15]. Such capability is particularly valuable for rapidly identifying early warning signals, enabling timely detection of potentially hazardous or catastrophic events before they escalate [14]. SB-UVPDs, compatible with next-generation devices, can be highly valuable for both military and civilian applications. A Ga_2_O_3_-based photodetector operating at various UV wavelengths is utilized in both military and civil applications, as illustrated in Figure 1 [16].

**Figure 2:**
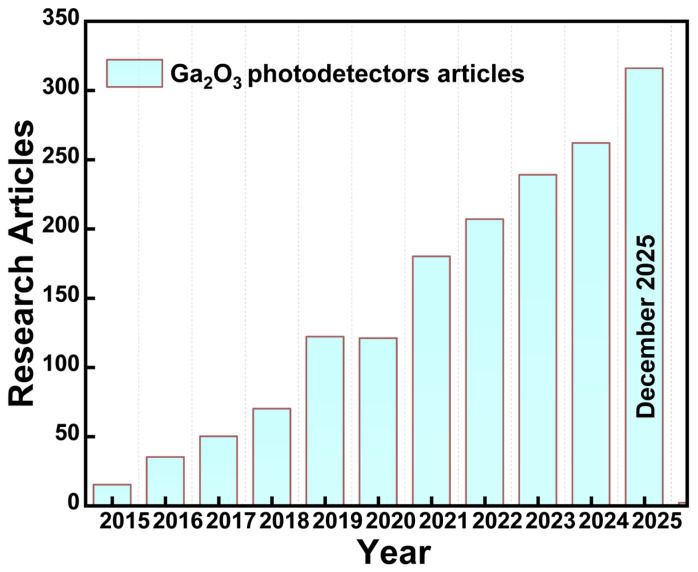
Number of published articles based on Ga_2_O_3_-based photodetectors from 2015 to December 2025; data collected from Web of Science Core Collections.

Available WBGs, such as silicon carbide (SiC), diamond (C), aluminum gallium nitride (AlGaN), and zinc oxide (ZnO), as well as zinc magnesium oxide (ZnMgO), have recently attracted considerable attention as a prominent choice for SB-UVPD [2,17]. However, photodetectors built with these wide-bandgap materials have generally performed less efficiently and have been limited in their performance. AlGaN-based SB-UVPD, in particular, has shown a decrease in performance as aluminum doping levels increase [18]. Achieving the targeted bandgap energy requires an Al content of approximately 40 atom%, a level of incorporation that can induce structural imperfections and defect formation [19]. Similarly, alternative WBGs, such as ZnMgO-based SB-UVPD, cannot generate an energy bandgap (up to 4.5 eV), which lowers device performance. In natural diamond-based SB-UVPD, the extremely wide bandgap of approximately 5.5 eV introduces several inherent limitations. These devices are primarily sensitive to wavelengths below 225 nm, which restricts their operational spectral range. Furthermore, the high material and fabrication costs, combined with significant challenges associated with controllable doping, hinder their widespread practical implementation [20,21,22]. Due to the inherent device performance limitations of conventional WBGs, including AlGaN, ZnMgO, and diamond used in SB-UVPD, the exploration of alternative materials with optimally tuned bandgap energies has become essential. Among emerging candidates, *β*-Ga_2_O_3_ has attracted considerable attention for SB-UVPD applications, owing to its favourable bandgap and superior optoelectronic properties, which enable enhanced device performance [23]. It consists of a wide energy bandgap range of (~4.6–4.9 eV) [24,25,26,27,28], a high melting point (1795 °C) [29], a high Baliga figure of merit (BFOM) (~3214.1) [29,30], a high electron mobility (~300 cm^2^ V^−1^ S^−1^) [29], a dielectric constant (~10–14.2) [31], breakdown electric field (~13 MV/cm) [32], and thermal conductivity (~0.11 Wcm^−1^ K^−1^) [29]. It is the most chemically and thermally stable polymorph of Ga_2_O_3_ among *α*, *γ*, *δ*, and *ɛ*-Ga_2_O_3_ [33,34]. It offers high tolerance to electric fields and effective control in harsh environments [15]. SB-UVPDs based on *β*-Ga_2_O_3_ have shown lower conduction losses, higher efficiency, faster response time, and high detectivity. Research on Ga_2_O_3_-based photodetectors has recently increased, as shown in Figure 2 [35].

In this context, this review explores recent progress in PVD-sputtered *β*-Ga_2_O_3_-based SB-UVPDs for next-generation optoelectronic devices. However, numerous review articles are available on Ga_2_O_3_-based SB-UVPDs—for instance, that by Chen et al. [11]—that have reviewed *β*-Ga_2_O_3_-based SB-UVPDs, focusing on growth, device fabrication, and applications. This article explored various growth techniques for fabricating *β*-Ga_2_O_3_ thin films. Also, they have explored photodetectors (PDs) of other structures, such as MSM (metal–semiconductor–metal) and Schottky junction PDs, i.e., MSM (metal–semiconductor–metal) junctions, field-effect transistor, and heterojunction-based PDs. Similarly, Kaur et al. [36] reviewed a Ga_2_O_3_-based SB-UVPD, highlighting its progress and prospects, and emphasized the essential performance aspects of SB-UVPD, such as dark current, photocurrent, PDCR (photo-to-dark current ratio), response time, etc., which are attributed to Ga_2_O_3_ properties. They also addressed the primary issues that tend to hinder device performance. And Xu et al. [8] reported Ga_2_O_3_-based SB-UVPDs, including PD architectures such as photoconductive, p-n junction, and Schottky junction devices. They reported the architectures of PDs, their individual advantages, and prospects for improvement. After brief research, they concluded that all device structures have their own pros and cons, depending on the requirements.

However, the primary focus on *β*-Ga_2_O_3_-based SB-UVPDs fabricated by PVD RF magnetron sputtering for next-generation devices remains unavailable. Therefore, in this review, we aim to explore progress in *β*-Ga_2_O_3_-based SB-UVPDs fabricated by PVD magnetron sputtering for next-generation PDs. This review will also cover the structure and performance characteristics of photodetectors and provide brief details on the operating parameters of the PVD magnetron sputtering method, *β*-Ga_2_O_3_, and the performance characteristics of *β*-Ga_2_O_3_-based SB-UVPDs for next-generation optoelectronics used in both civilian and military contexts.

This review is organized into three main sections. Section 1 provides an overview of SB-UVPDs and their device performance metrics, introduces the fundamental properties of *β*-Ga_2_O_3_, and outlines the operating principles of PVD RF magnetron sputtering, with an emphasis on how deposition parameters influence thin-film material properties. Section 2 focuses on the morphological and structural characteristics of *β*-Ga_2_O_3_-based SB-UVPDs fabricated via RF magnetron sputtering. Section 3 examines the device performance characteristics of *β*-Ga_2_O_3_-based SB-UVPDs, highlighting their potential for integration into next-generation optoelectronic applications.

## 2. Overview of Solar-Blind UV Photodetector (SB-UVPD), *β*-Ga_2_O_3_, and PVD RF Magnetron Sputtering Method

### 2.1. SB-UVPDs and Their Performance Characteristics

**Figure 3:**
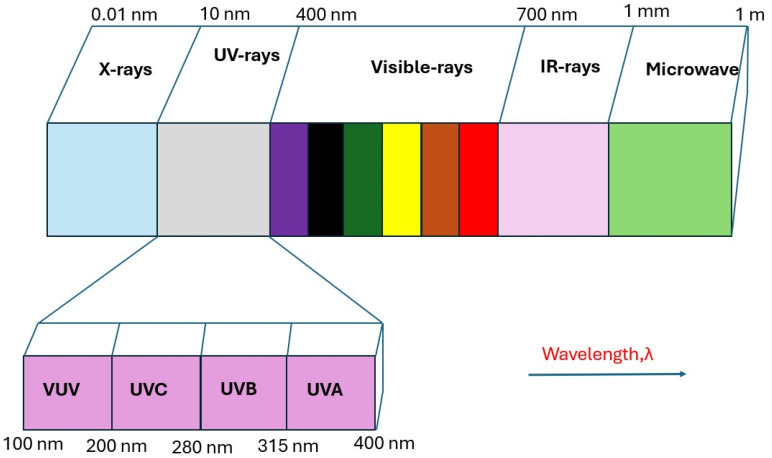
Electromagnetic spectrum: screening ultraviolet rays (UVA, UVB, UVC and EVC), the visible region, and the infrared.

The wavelength range of the UV spectrum between 200 and 280 nm is referred to as the solar-blind region [5], as shown in Figure 3 [37].

An SB-UVPD is an important optoelectronic device designed to selectively sense ultraviolet radiation within the solar-blind spectral range and convert the incident photon flux into a corresponding electrical signal, while effectively suppressing interference from visible and solar radiation [10]. The operation of the SB-UVPDs is governed by three fundamental mechanisms, i.e., [36].

1. Electron–hole pairs generation upon absorption of suitable incident photons;
2. Transport of the photogenerated carriers through the active region, which may involve carrier multiplication via internal gain processes;
3. Charge carrier collections at the electrodes, resulting in the measurable electrical output signal.

**Figure 4:**
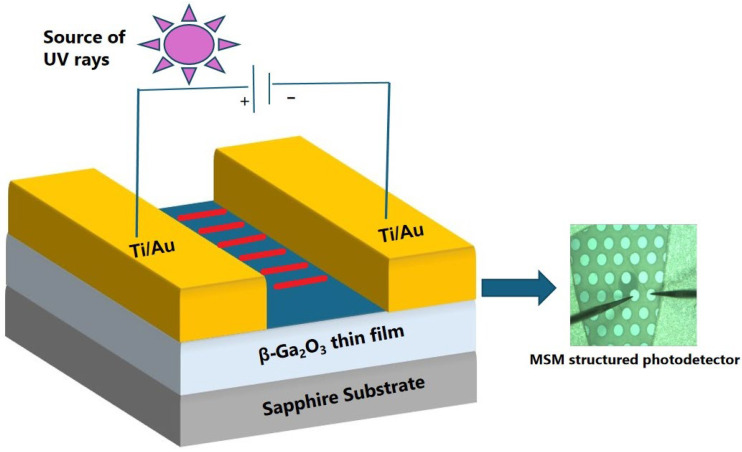
Schematic diagram of *β*-Ga_2_O_3_-based solar-blind UV photodetector of M-S-M structure.

It converts incident solar-blind UV light into a quantifiable electrical output through a series of stages. First, photons absorbed in the device’s active layer form electron–hole pairs, known as photo-generated carriers. These carriers then flow through the device, potentially amplified by techniques like avalanche multiplication to increase signal intensity. In the final stage, the airlines are gathered at the electrodes, allowing the absorbed optical energy to be converted into an electrical signal that may be recognized, quantified, and used for further research or applications. Figure 4 shows the typical *β*-Ga_2_O_3_-based SB-UVPDs of the MSM structure [37]. A MSM photodetector device features two Schottky metallic contacts (platinum/gold) in an interdigitated electrode arrangement. During device operation, an external bias is applied across the electrodes to establish an internal electric field. Upon UV illumination at 254 nm or 365 nm, electron–hole pairs are generated in the semiconductor region between the electrodes, which are subsequently separated and driven by the electric field toward the respective electrodes, giving rise to a measurable photocurrent. The quality of the Schottky contact significantly impacts device performance.

It serves as a fundamental element in optoelectronic systems by converting the energy carried by incident photons into a corresponding electrical signal through photoelectric processes [38]. It is widely used across both defence and civilian sectors, with key applications including missile tracking and guidance systems, navigation technologies, advanced optical imaging, and space-based optical communication platforms.

#### 2.1.1. Performance Characteristics of Solar-Blind Photodetectors

**Figure 5:**
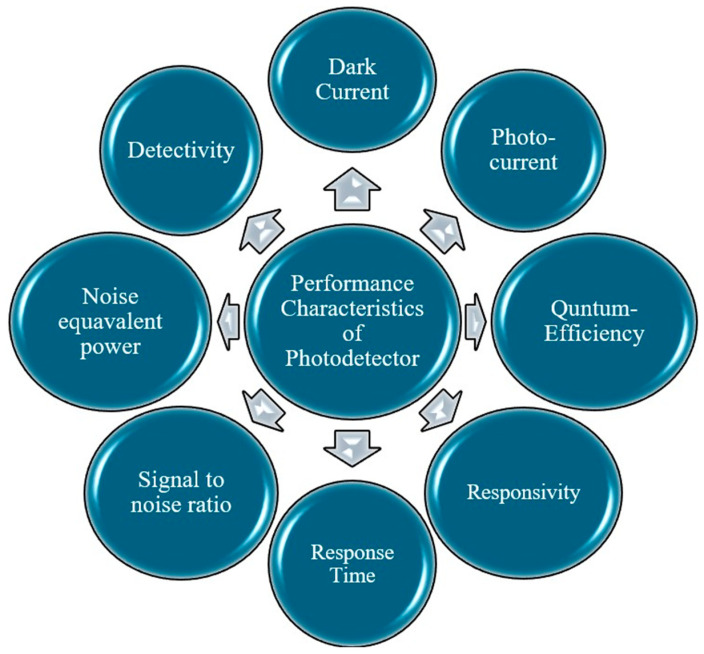
Performance characteristics of a photodetector.

The device performance of a PD is valued through the listed performance characteristics, as mentioned in Figure 5 [17,39].

##### Dark Current (I_dark_)

Dark current is a small current that passes through the photodetector in the absence of photons [36]. It is composed of the semiconductor junction’s saturation current and radiation current. It is often associated with specific faults in the depletion layer, partially reflecting device selectivity.

##### Photo Current (I_photo_)

An excited electron creates an electron–hole pair when an intense photon hits the photodetector. Assume that the depletion layer is where photon absorption takes place. Photocurrent is created when the internal electric field in this area removes the barrier between electrons and holes, allowing the latter to flow towards the cathode and the former to flow towards the anode [8].

##### Quantum Efficiency

Quantum efficiency refers to a photodetector’s ability to convert input light into an electrical signal [17]. It measures a device’s sensitivity to optical radiation. These are mainly (1) internal quantum efficiency (IQE) and (2) external quantum efficiency (EQE). A semiconductor’s IQE is defined as the ratio of the number of electron–hole pairs produced to the number of incident photons per second. EQE is the ratio of electron–hole pairs gathered to incident photons per second. EQE is typically considered for gallium oxide-based solar-blind photodetectors [36]. It is expressed as in Equation (1):

(1) $$EQE=\frac{Rhc}{e\lambda }$$

*R* represents responsivity, *c* is the speed of light, *h* is Planck’s constant, *e* is the electronic charge, and *λ* is the wavelength of photons.

##### Responsivity

Responsivity is an important photodetector metric, defined as the ratio of the photocurrent generated to the incident optical power. It expresses how efficiently the device converts incoming photons into an electrical current, often measured in amperes per watt (A/W) [17].

(2) $$R=\frac{I_{photo}-I_{dark}}{Indicent\;optical\;Power}$$

##### Response Time

The response time of a photodetector defines how quickly its output adapts to variations in incident light intensity and is composed of two key components.

I. Rise time: The interval required for the detector’s output to increase from 10% to 90% of its peak value upon illumination.
II. Decay time: The interval during which the output decreases from 90% to 10% of its maximum value after the light is removed.

##### Signal-to-Noise Ratio (SNR)

The SNR quantifies the ratio of the desired signal to the background noise. It is defined mathematically as the ratio of signal power to noise power, providing a measure of the detector’s ability to distinguish the signal from unwanted fluctuations. The following equation expresses SNR (3):

(3) $$SNR=10log\frac{P_{signal}}{P_{noise}}$$

##### Specific Detectivity (D*)

Specific detectivity is a figure of merit for photodetectors that measures their ability to distinguish weak optical signals from background noise [17]. It normalizes detectivity based on detector area and measurement bandwidth, enabling device comparisons. Higher numbers indicate greater sensitivity and improved performance, though they are limited by noise. It is expressed as in Equation (4):

(4) $$D^{*}=R\cdot \sqrt{\frac{A_{OP}}{2.e.I_{dark}}}$$

Here, *A_op_* is the effective area under illumination.

### 2.2. Photodetector Architecture

This section will discuss the most used photodetector architecture [40].

#### 2.2.1. p-n Junction Photodiode

**Figure 6:**
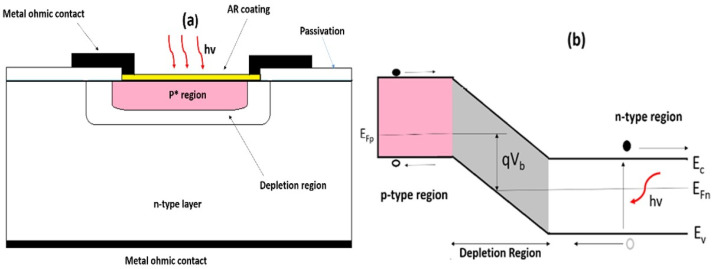
(**a**) Structural configuration and (**b**) band diagram of p-n junction photodiode. Adapted from ref. [40]. CC by 4.0.

In a p-n photodiode, a shallow p-type region is diffused into an n-type active layer. Photons exceeding the semiconductor’s bandgap energy strike neutral regions on either side of the junction, generating electron–hole pairs. These carriers diffuse toward the junction’s space charge region, where the built-in electric field separates them. Minority carriers on one side convert to majority carriers on the other. The resulting photocurrent produces a voltage in open-circuit conditions or drives current flow under short-circuit operation. The device’s structure and band diagram are shown in Figure 6.

#### 2.2.2. Metal–Insulator–Semiconductor (M-I-S) Photodiodes

**Figure 7:**
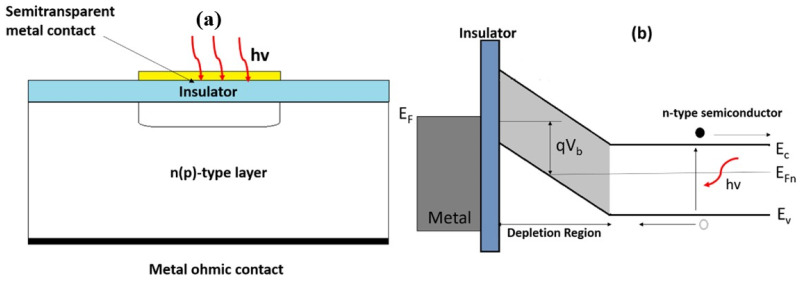
(**a**) Structural configuration and (**b**) band diagram of metal–insulator–semiconductor (MIS) photodiodes. Adapted from ref. [40]. CC by 4.0.

An M-I-S photodiode uses an insulating layer to separate a metal gate from the semiconductor surface. For proper operation, this insulator must be at least 10 nm thick—thin layers can lead to electron quantum tunnelling. When negative voltage is applied to the metal electrode, it pushes electrons away from the insulator–semiconductor interface, creating a depletion region. The photogate’s well capacity measures the maximum charge it can hold. Capacitance depends on several factors, including gate polarization, insulator thickness, electrode area, and the semiconductor’s background doping level. The structure and band diagram are illustrated in Figure 7 [40].

#### 2.2.3. Schottky Barrier Photodiodes

**Figure 8:**
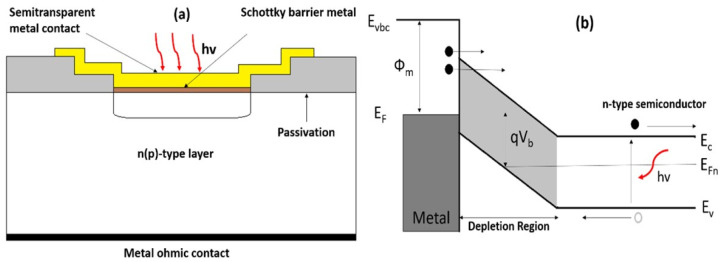
(**a**) Structural configuration and (**b**) band diagram of Schottky barrier photodiode. Adapted from ref. [40]. CC by 4.0.

A Schottky barrier photodiode operates based on an M-S junction. Here, E_c_ and E_v_ represent the conduction and valence bands, while E_f_ and Φ_m_ denote the metal’s Fermi level and work function. Like a p-n junction, the M-S interface creates a barrier that separates optically generated electron–hole pairs. Carrier generation occurs in both the neutral semiconductor region and the space-charge region at the M-S boundary. Schottky barriers offer key advantages over other photodiodes, including simple fabrication by depositing metal onto n- or p-type semiconductors, eliminating the need for high-temperature diffusion, and providing quick response times. However, they tend to have a relatively high dark current. The structure and band diagram are shown in Figure 8 [40].

#### 2.2.4. Avalanche Photodiodes (APDs)

**Figure 9:**
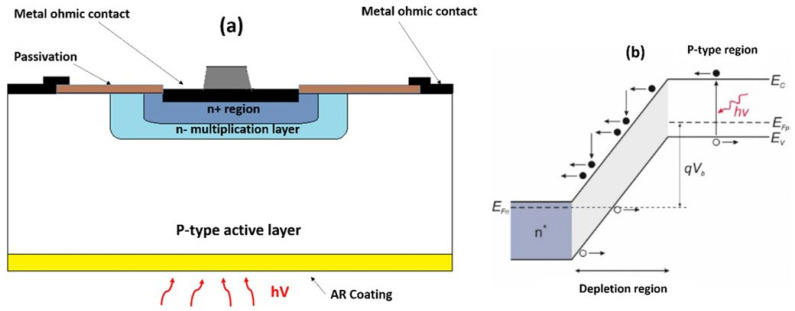
(**a**) Structural configuration and (**b**) band diagram of Avalanche photodiodes (APDs). Adapted from ref. [40]. CC by 4.0.

An APD perceives small, weak optical signals using carrier amplification. Apply a sufficient reverse bias to the device to ensure the depletion layer spans the whole n-region. Photon absorption occurs in the p-region. When photogenerated minority carriers, such as electrons, approach the boundary of the depletion zone, they are driven by an electric field and accumulate enough energy to begin multiplication. APD provides excellent speed, sensitivity, and quantum efficiency. The structural configuration and band diagram are shown in Figure 9 [40].

#### 2.2.5. PIN Photodiodes

**Figure 10:**
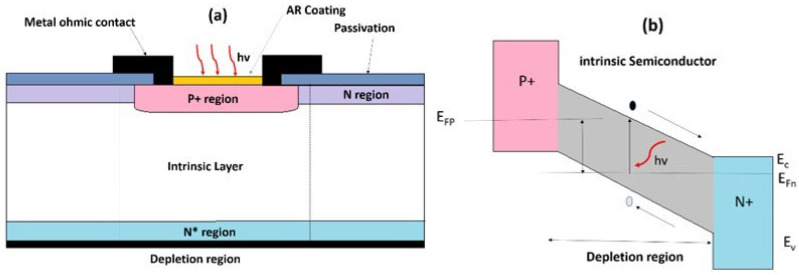
(**a**) Structural configuration and (**b**) band diagram of PIN photodiodes. Adapted from ref. [40]. CC by 4.0.

A PIN photodiode is a conventional photodiode with an intrinsic region (absorber) between the p-type and n-type. This region is often heavily doped to achieve an ohmic contact. With the correct reverse bias, the depletion area occupies the entire intrinsic volume of the absorber, given by the product of the absorber’s doping thickness [40]. When the energy gap between p- and n-regions is larger, incident photons are absorbed solely in the depletion zone, forming e-h pairs. The absence of an electrically neutral volume results in no diffusion current in the device. Furthermore, the dark current is caused by defect centres in the diode depletion zone, which generate minority carriers. Although diffusion dark current is generated in neutral p- and n-type regions, it may be minimal if the energy gap is large. The structural configuration and band diagram are shown in Figure 10 [40].

The most studied type of photodetectors, SB-UVPD, continues to receive technological developments in next-generation devices. Researchers, scientists, and engineers continue to work on enhancing the sensitivity, response time, and durability of these detectors.

### 2.3. β-Gallium Oxide (Ga_2_O_3_)

**Figure 11:**
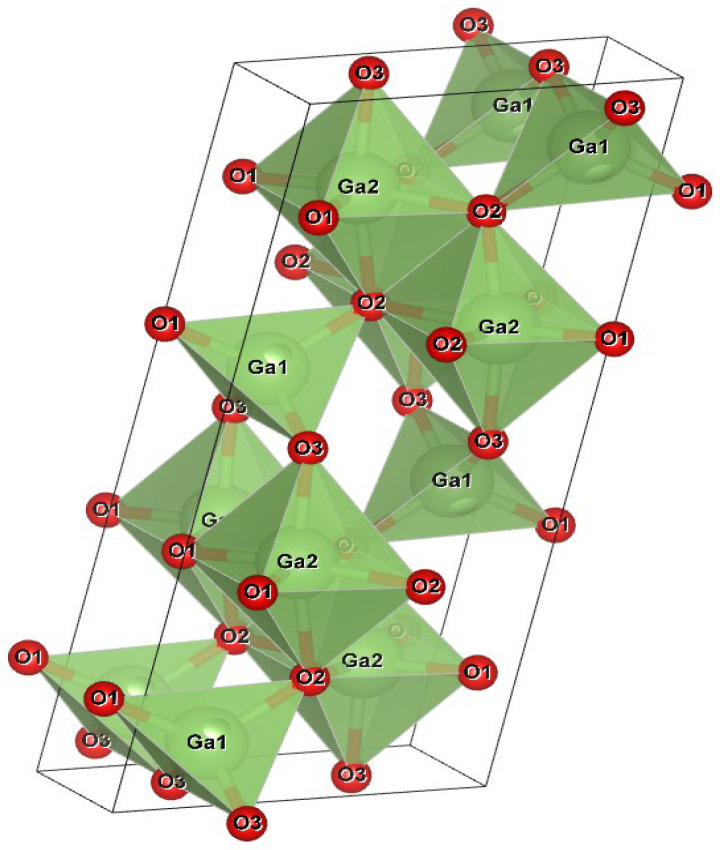
Crystal unit structure of *β*-Ga_2_O_3_, exported from Vesta Software (version 3.90.1a).

Despite over seven decades of research and development, Ga_2_O_3_ has recently gained recognition as a semiconductor material [41,42]. Historically, silicon-based technology has dominated the semiconductor industry for electronics, power devices, and optoelectronics, yet it has persistently fallen short of meeting the ever-growing performance demands [43]. Nowadays, WBGs such as *β*-Ga_2_O_3_, GaN, SiC, diamond, AlGaN, ZnO, and ZnMgO have the potential to replace Si-based technology and are performing better. These wide-band semiconductors have been effectively manufactured, which has a significant impact on our daily lives. However, the manufacturing of these semiconductors is impeded by technological challenges in obtaining large quantities of high-quality crystals. Among all WBGs, *β*-Ga_2_O_3_ exhibits beneficial properties and can be grown substantially. It is one of the stable polymorphs of Ga_2_O_3,_ as shown in Table 1. Figure 11 illustrates the unit structure of *β*-Ga_2_O_3_, along with its theoretically studied band structure [35]. Moreover, Table 2 compares the essential characteristics of *β*-Ga_2_O_3_ with those of other wide-bandgap semiconductors.

### 2.4. PVD RF Magnetron Sputtering Method

RF sputtering is a versatile physical vapour deposition (PVD) technique widely used to fabricate thin films of insulating, semiconducting, and metallic materials. In this method, a plasma is generated by applying an alternating electric field, typically at 13.56 MHz, between a target electrode and the substrate chamber [46,47]. To date, several deposition methods have been employed to deposit Ga_2_O_3_ as a thin film, including the pulse laser deposition technique (PLD) [48,49,50], molecular beam epitaxy (MBE) [51,52], metal–organic chemical vapour deposition (MOCVD) [53,54,55], physical vapour deposition (PVD), RF magnetron sputtering [56,57,58,59], low-pressure chemical vapour deposition (CVD) [60,61], mist CVD [62,63], sol–gel method [64,65,66], etc., for the fabrication of *β*-Ga_2_O_3_ as thin films. However, among the methods mentioned above, RF sputtering, LPCVD, PLD, and MBE produced the highest-quality results. Nevertheless, the PLD, MBE, and LPCVD methods are limited to small-scale, non-uniformity, to large-area, low-speed manufacturing, and to smaller deposition areas [67]. As a result, the PVD RF sputtering method may be the best alternative method for fabrication because it can produce high-quality thin films at a high deposition rate and large deposition area with uniformity by controlling working parameters such as sputtering power, deposition time, gas pressure, chamber pressure, substrate temperature, and post-annealing treatment, among others [67,68,69].

#### 2.4.1. Working Principle

**Figure 12:**
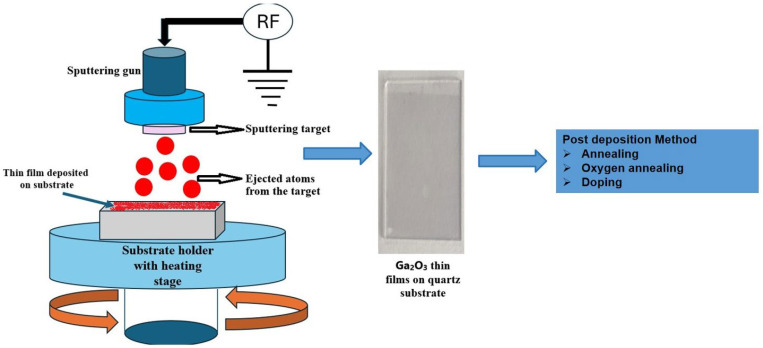
Schematic diagram of the PVD RF sputtering deposition method.

Radio frequency (RF) sputtering is a rapid vacuum coating technique used to deposit metals, alloys, and compounds on various materials, with thicknesses ranging from nanometres to millimetres [70]. In this method, the target materials and cleaned substrate are initially attached to the chamber, and a chamber pressure in the 10^−6^ to 10^−8^ mbar range is to be maintained [71]. As argon (Ar) gas is introduced into a vacuum chamber, charged electrons accelerate neutral Ar to generate Ar^+^, which is propelled to the target (cathode) and strikes the surface. As a result, atoms become loosened from the target material and begin to eject and start to deposit on the substrate one by one [71,72,73]. Finally, as per the requirements, these fabricated samples are subjected to post-processing methods, such as annealing treatment and doping, to tune their optical and electrical properties, as shown in Figure 12, which displays the schematic diagram of the RF sputtering process for depositing a thin film on a substrate.

#### 2.4.2. Working Parameters

This method has key working parameters such as sputtering power (W), deposition duration, substrate temperature, gas flow rate (SCCM), substrate orientation, etc., that are crucial for producing thin films at their best performance levels [68,74]. Below, the impact of these factors on the structural, optical, and electrical aspects of thin films is examined.

##### Sputtering Power

RF sputtering power is crucial in creating consistent, uniform, and adherent thin films. It plays a vital role in producing thin films with improved crystallinity and a consistent deposition rate for device manufacturing. Researchers have fabricated gallium oxide thin films using various sputtering techniques. Some researchers fabricated Ga_2_O_3_ thin films using the sputtering power ranging from 160 W to 200 W. They found that increasing the sputtering power tends to enhance both the grain size and the crystallinity of the Ga_2_O_3_ thin film [75,76]. The larger sputtered particles, driven by higher energy, enable higher crystallinity by facilitating migration to more suitable lattice sites. Researchers also observed that the thin-film deposition rate increases with increasing sputtering power [77]. The optical energy bandgap sometimes decreased when the sputtering power was increased [78].

##### Deposition Time

Sputtering power and deposition time have very similar effects on the properties of deposited thin films. Increasing the deposition time enhances the thickness and crystallinity of gallium oxide thin films. Researchers reported that increasing deposition time enhanced the crystallite size and crystallinity of the thin films produced [79,80]. Deposition time can be crucial for improving structural properties with adjustable thin-film thickness.

##### Substrate Temperature

The substrate temperature has a significant impact on numerous thin-film characteristics. It can play a substantial role in improving thin-film crystallinity and electrical properties. The substrate temperature affects the thin film’s grain size by improving its nucleation and growth kinetics. Ga_2_O_3_ thin films with stoichiometric Ga and O concentrations could only be deposited at 300 °C or higher temperatures [81]. Additionally, some researchers have observed that thin-film grain size increases with increasing substrate temperature [82]. The electrical mobility in thin films is observed to increase with increasing substrate temperature [83].

##### The Flow of Gases (Argon/Oxygen/Nitrogen Gas)

Another crucial parameter in the deposition process is the gas flow during RF sputtering. Under controlled conditions, a gas mixture (with an oxygen-to-argon or nitrogen ratio) must be selected to achieve the desired thin-film crystallinity and high deposition rate. The gas flow rate has been found to significantly influence the deposition conditions during RF sputtering. Increasing the working gas flow rate is thought to improve the energy flux at the substrate, resulting in faster deposition and larger grain development [84].

##### Substrate Orientation

The substrate’s orientation is critical in determining the thin film’s characteristics. When selecting the substrate and coating, the ideal approach is to minimize lattice mismatch. Substrate orientation can also impact the morphological and structural properties. Kaur et al. [85] reported on fabricating *β*-Ga_2_O_3_ thin films on different-oriented (100), (110), and (111) silicon substrates. They observed that the grain size for Si (100) was 22.4 nm smaller than for Si (110), and for Si (111) this was 36.6 nm and 40.6 nm, respectively.

##### Elemental Doping

The properties of a thin film can be significantly altered by selectively introducing different elements into the pure semiconductor. Dopant materials can affect thin-film structural properties, such as crystallinity and grain size. Dopant materials, such as aluminum (Al), the rare-earth europium (Eu), tin (Sn), and silicon (Si), can influence the structural, optical, and electrical properties of Ga_2_O_3_ thin films. The bandgap increased from 4.9 to 6.1 eV with increasing Si doping concentration (0–50%), as reported by Takakura et al. [86], while adding Sn to *β*-Ga_2_O_3_ decreased the bandgap energy relative to the intrinsic *β*-Ga_2_O_3_, as reported by Kudou et al. [87].

##### Post-Annealing Temperature

The post-annealing temperature is crucial for achieving the crystalline structure of thin films produced by RF sputtering. It significantly enhances thin-film quality by reducing roughness. It substantially impacts the optical and electrical characteristics of thin films. It significantly affects the structural, optical, and electrical properties of thin Ga_2_O_3_ films. Researchers fabricated a Ga_2_O_3_ thin film and post-annealed the sample, observing a transition in the Ga_2_O_3_ structure from amorphous to crystalline as the annealing temperature increased [88,89]. The peak intensity increased with higher annealing temperature. It was also found that the grain size of the thin film improved in different annealing environments [90]. The post-annealing temperature has a significant impact on the Ga_2_O_3_ phases. As-deposited thin film post annealed at 400 °C was observed to be *γ*-Ga_2_O_3_, but when the annealing temperature was increased from 600 °C to 1000 °C, it was observed to be monoclinic *β*-phase Ga_2_O_3_ [91,92]. Moreover, the surface roughness of Ga_2_O_3_ thin films was also influenced by post-annealing temperature. The thin film’s surface roughness improved as the annealing temperature increased. On the other hand, the optical bandgap increased up to 800 °C but decreased when annealed at higher temperatures (>1000 °C).

## 3. Review Based on PVD RF Magnetron-Sputtered *β*-Ga_2_O_3_-Based SB-UVPD

This section will review the PVD RF magnetron-sputtered *β*-Ga_2_O_3_-based SB-UVPD, focusing on morphological, structural, and device performance, as well as the key working parameters of the PVD magnetron sputtering method. Therefore, for review, we will categorize the section into three parts: the first will discuss morphological and structural properties, the second will discuss optical properties, and the third will focus on device performance characteristics (electrical properties).

### 3.1. Morphological and Structural Properties

Morphology and structural properties are crucial in thin-film technology, as they influence a material’s optical, electrical, and mechanical properties. Morphological analysis is a branch of analytical imaging, a complex type of high-resolution imaging that employs sophisticated microscopes to produce photographs of the surfaces of materials invisible to the human eye. Before fabricating any devices, researchers must undergo morphological analysis of thin films to achieve optimal performance. Regarding structural properties, terms such as phases, crystal structure, grain size, energy bandgap, conductivity, corrosion resistance properties, lattice defects, etc., are crucial for device performance. It directly impacts the optical, electrical, and mechanical properties of any material. This tuning is essential for achieving optimal performance in thin-film semiconductor devices. Here, we will review the morphological and structural properties, considering PVD magnetron sputtering working parameters such as sputtering power, post-annealing temperature, deposition time, substrate orientation, and film thickness.

#### 3.1.1. Based on Post-Annealing Temperature

**Figure 13:**
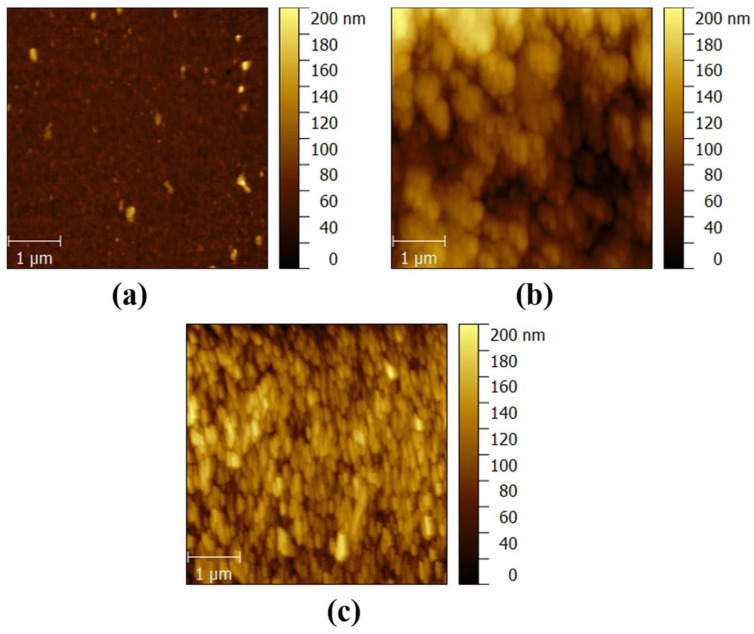
AFM micrograph of (**a**) as-deposited Ga_2_O_3_, annealed samples at (**b**) 800 °C, and (**c**) 900 °C. Adapted from ref. [95]. CC by 4.0.

**Figure 14:**
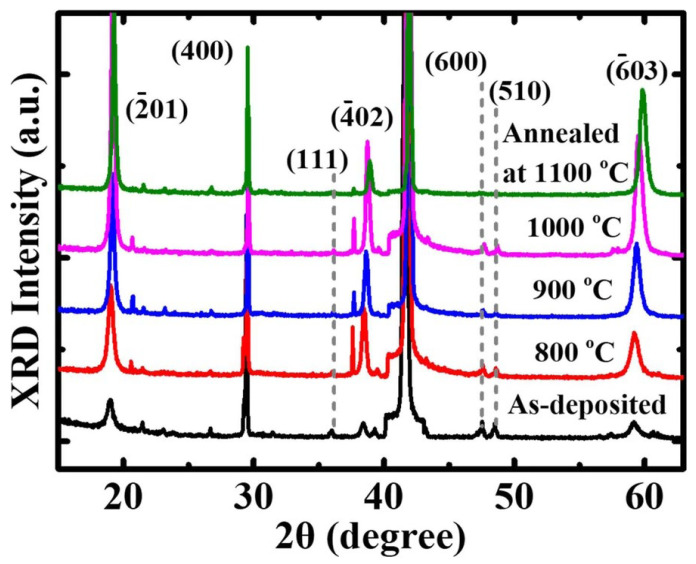
XRD peak of as-deposited and annealed samples at (800 °C, 900 °C, 1000 °C, and 1100 °C). Adapted from ref. [95]. CC by 4.0.

Post-annealing is considered the final step in the PVD magnetron sputtering method for improving the strength, phase, and crystallinity of thin films for a device [93,94]. It evolves to correcting the stoichiometry, grain growth, and oxygen vacancy defects within the Ga_2_O_3_ thin film. As is well known, thin films deposited by RF sputtering generally form an amorphous structure; however, they can also form a polycrystalline structure [56]. Numerous articles are available on the impact of annealing treatment on Ga_2_O_3_ for solar-blind UV photodetectors. Here, we will discuss a selection of published articles. For example, Hu et al. [95] reported the morphological and structural properties of a Ga_2_O_3_ thin film fabricated using a substrate temperature of 600 °C and later post-annealed at 800 °C, 900 °C, 1000 °C, and 1100 °C, respectively, for Ga_2_O_3_-based SB-UVPD. Furthermore, the morphological and structural properties of the samples were studied using AFM and XRD. According to the AFM results, the surface roughness RMS value of both as-deposited and annealed samples increased with increasing temperature; moreover, the grain size of the samples also increased, as indicated in Figure 13. Additionally, the structural properties of all samples were investigated by XRD, and the results showed that the XRD peak intensity increased upon annealing compared to the as-deposited Ga_2_O_3_ thin films, as shown in Figure 14. Grain size was also reported to increase slightly in as-deposited and annealed samples (800 °C to 1000 °C), but to decrease in annealed samples at 1100 °C.

**Figure 15:**
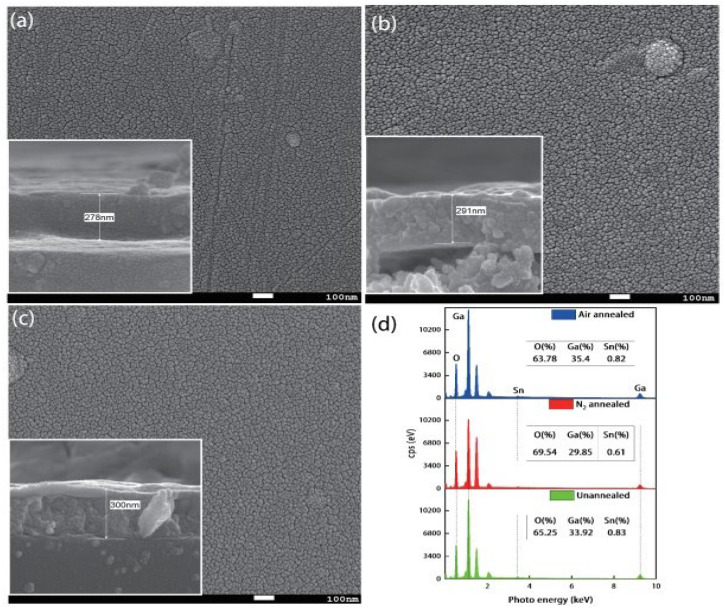
(**a**–**c**) The FESEM micrograph of the as-deposited Sn-doped Ga_2_O_3_ and annealed sample in nitrogen and air atmosphere, and (**d**) EDX result. Adapted with permission from ref. [96]. Copyright © 2024. IOP Publishing.

**Figure 16:**
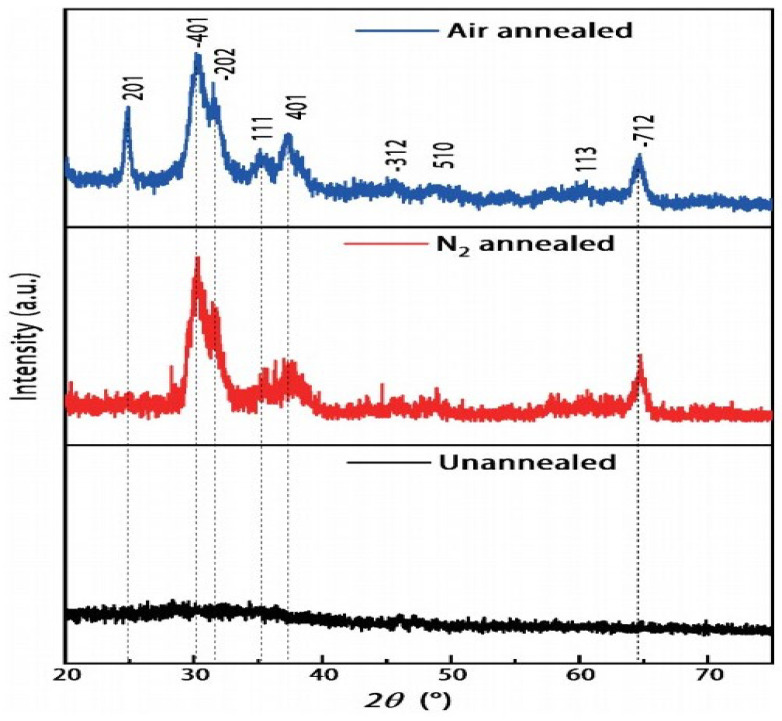
XRD graph of Sn-doped Ga_2_O_3_ sample and annealed sample. Adapted with permission from ref. [96]. Copyright © 2024. IOP Publishing.

Similarly, Li et al. [96] reported a Sn-doped Ga_2_O_3_-based SB-UVPD fabricated by RF magnetron sputtering. The experimental parameters used in this method were a sputtering power of 140 W, an argon gas flow rate of 40 SCCM, and a deposition time of 2 h, respectively. Furthermore, the deposited thin film was subsequently annealed at 900 °C for 2 h in both nitrogen and air atmospheres. Upon analysis, they reported that the as-deposited Sn-doped Ga_2_O_3_ sample had tiny particles on its surface, as observed by FE-SEM (Figure 15). However, upon annealing the sample in a nitrogen-air atmosphere, these tiny particles were eliminated, and the uniformity of the thin film was improved. Furthermore, XRD was conducted for the structural property analysis, where they reported that the as-deposited Sn-doped Ga_2_O_3_ consisted of an amorphous nature and had no XRD peak. In contrast, the samples annealed in nitrogen and air atmospheres were reported to be in a crystalline form, assigned to the *β*-Ga_2_O_3_ phase as shown in Figure 16.

Similarly, Wang et al. [94] reported the impacts of annealing temperature on the morphological and structural properties of Ga_2_O_3_-based SB-UVPD. They report morphological and structural properties by depositing Ga_2_O_3_ thin films and subsequently annealing the samples at 500 °C, 700 °C, and 900 °C, respectively. The XRD analysis reported that the as-grown samples at room temperature were amorphous, while the samples annealed at 500 °C, 700 °C, and 900 °C were reported to be nanocrystalline, corresponding to the β-phase of Ga_2_O_3_. Also, the FWHM was reported to decrease with increasing annealing temperature, while the crystal size (d) was shown to increase.

#### 3.1.2. Based on Substrate Temperature

Another important operating parameter of PVD magnetron sputtering, i.e., substrate temperature, has a significant impact on thin-film growth, crystallinity, and defect formation. Lower substrate temperatures tend to result in poor-quality thin films, such as amorphous or polycrystalline thin films. A higher substrate temperature tends to cause thin-film cracking; therefore, a moderate substrate temperature is essential for producing well-uniform thin films and a crystalline form. It evolves during the fabrication of a Ga_2_O_3_ thin film for a device (SB-UVPD) to achieve well-controlled stoichiometry and a highly crystalline form. Some of the available articles have been reviewed regarding the impact of substrate temperature on Ga_2_O_3_ thin films.

Researchers Chen et al. [59] fabricated Ga_2_O_3_ thin films for an SB-UVPD using substrate temperatures of 600 °C, 650 °C, and 700 °C, respectively. They reported that all samples were uniform and smooth in appearance. Moreover, XRD analysis indicated that the sample deposited at 600 °C exhibited very weak crystallinity, attributed to the monoclinic β-phase of Ga_2_O_3_. However, when the annealing temperature was increased to 650 °C, peaks corresponding to planes (400) and (800) began to appear, and the thin film’s crystallinity improved. At 700 °C, both peaks at planes (400), (600), and (800) were visible and were of high crystalline form. Similarly, Cui et al. [97] reported structural properties of *β*-Ga_2_O_3_ thin film fabricated for *β*-Ga_2_O_3_-based SB-UVPD by the PVD magnetron sputtering method. XRD further analyzed the structural properties; the XRD peaks observed in all samples were assigned to *β*-Ga_2_O_3_ at the planes (−201), (−402), and (−603), respectively, oriented with respect to the (−201) planes. The intensity of the XRD peak was reported to increase upon increasing the growth temperature during the fabrication of *β*-Ga_2_O_3_ thin films. Moreover, the FWHM of the grown *β*-Ga_2_O_3_ thin film decreased until the growth temperature reached 650 °C, but it suddenly increased at 700 °C.

#### 3.1.3. Based on Film Thickness

Film thickness is a crucial factor in PVD RF sputtering, as it significantly influences the surface morphology and structural properties of the deposited material. Very thin films fabricated by PVD RF sputtering often contain several defects, including surface pinholes, amorphous regions, and, occasionally, island growth. In contrast, an intermediate range of film thickness improves film crystallinity and uniformity, whereas thick films cause cracking or peeling from the substrate. Therefore, an optimized intermediate thin film thickness is necessary for a device (SB-UVPD) to achieve optimal results. Based on film thickness, a few articles are available on Ga_2_O_3_-based solar-blind UV photodetectors.

**Figure 17:**
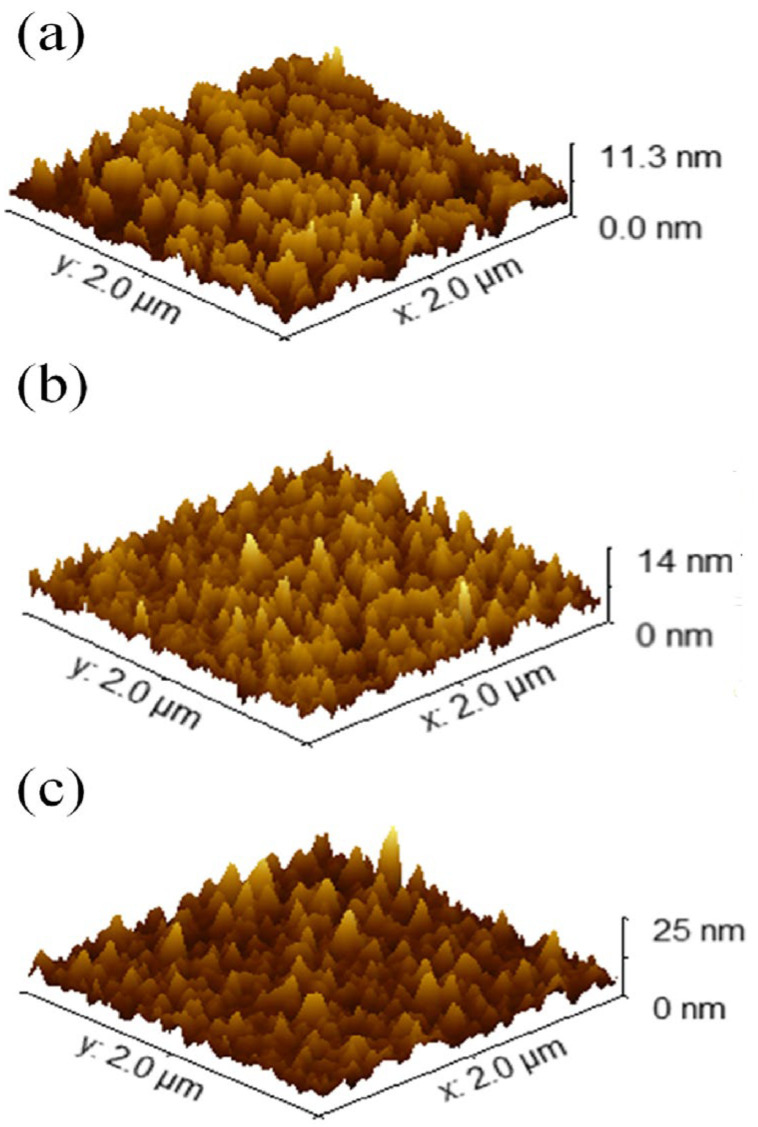
AFM micrograph of samples with (**a**) 20 nm, (**b**) 70 nm, and (**c**) 220 nm thicknesses. Adapted from ref. [13]. CC by 4.0.

Tran et al. [13] reported a surface topography of Ga_2_O_3_-based SB-UVPDs fabricated through PVD RF magnetron sputtering using the AFM method. For the deposition of Ga_2_O_3_ thin film, they set the sputtering power to 100 W, the substrate temperature to 800 °C, and the chamber pressure to 3 mTorr, respectively. The surface topography of Ga_2_O_3_ thin film, reported for different thicknesses such as 20 nm, 70 nm and 220 nm, is shown in Figure 17. The RSM value reported an increase, i.e., from 1.34 nm RSM of 20 nm films to 2.71 nm of 220 nm thick films.

Similarly, Kaur et al. [39] reported that for *β*-Ga_2_O_3_-based SB-UVPD, varying film thickness impacts the morphological and structural properties. They fabricated film thicknesses of 5 nm, 20 nm, 50 nm, 100 nm, and 300 nm, respectively, for investigation using substrate temperature of 500 °C and sputtering power of 100 W. Morphological properties were analyzed using AFM, where they reported that root mean square roughness (RMS) was in the lower range for the sample with a film thickness of 5 nm and 20 nm, but RMS tends to increase for higher film thickness. The RMS values of 5 nm, 20 nm, 50 nm, 100 nm, and 300 nm film thickness samples were reported as 0.23 ± 0.04 nm, 0.23 ± 0.01 nm, 0.59 ± 0.03 nm, 0.68 ± 0.01 nm, and 1.12 ± 0.12 nm, respectively. Furthermore, the XRD results for structural analysis indicate that a characteristic peak at 2θ = 37.4° was observed for each sample, assigned to *β*-Ga_2_O_3_. However, continuity of the XRD peaks across the samples (5 nm, 20 nm, 50 nm, 100 nm, and 300 nm film thickness) was lacking, suggesting that the structural properties were affected by film thickness.

#### 3.1.4. Based on Sputtering Power

This is the most critical parameter in PVD magnetron sputtering, as it plays a crucial role in the fabrication of thin film devices (SB-UVPDs). It significantly impacts the growth and structural, optical, and electrical properties of Ga_2_O_3_ thin film. According to the literature review, lower sputtering power tends to form thin films as amorphous, porous, non-uniform, and sometimes nanocrystalline structures, while higher sputtering power tends to form thin films as highly crystalline, well-adhered, and controlled-composition structures [98]. Based on the effects of sputtering power on Ga_2_O_3_ thin film-based SB-UVPD, several articles have been reported. For instance, the research by Wang et al. [77] reported the impacts of sputtering power on the morphological and structural properties of *β*-Ga_2_O_3_-based SB-UVPDs. They varied the sputtering power to 60 W, 100 W, and 150 W and later annealed these as-deposited samples at 900 °C. They reported that the sputtering power significantly affected the film thickness and growth rate of *β*-Ga_2_O_3_ thin films. The thickness and growth rate of the thin films increased with increasing sputtering power. Additionally, they found that the surface of the *β*-Ga_2_O_3_ thin films was very rough at low sputtering powers (60 W and 100 W). However, at high sputtering power (150 W), the surface quality of the thin films improved. Moreover, the effects of sputtering power on structural properties were further analyzed by XRD, where it was reported that all deposited samples fabricated under different sputtering conditions were preferentially oriented towards the (−201) planes and were assigned to the monoclinic *β*-Ga_2_O_3_ phase. Similarly, Li et al. [99] reported the impact of sputtering power (80 W, 100 W, 120 W, and 140 W) on the thin film morphological properties of Ga_2_O_3_-based SB-UVPDs. They concluded that the thickness of thin films increases with increasing sputtering power (80–140 W). Moreover, grain size measurements showed an increasing trend in grain size of samples deposited with sputtering power from 80 W to 120 W, but showed a decreasing trend for the sample deposited at 140 W. Higher sputtering (140 W) power might induce defects in the thin film. They also reported the impacts of sputtering power on the structural properties of all samples through XRD analysis. They noted that only one peak was observed for all samples, which was preferentially oriented to the (−201) peak of the β-phase of Ga_2_O_3_.

#### 3.1.5. Based on Orientations of Substrate

Kaur et al. [85] reported a *β*-Ga_2_O_3_-based SB-UVPD fabricated via the PVD magnetron sputtering method on silicon substrates of different orientations, i.e., Si (100), Si (110), and Si (111). They used a sputtering power of 100 W, substrate temperature of 750 °C, and base vacuum chamber pressure of 8 × 10^−7^ Torr. *β*-Ga_2_O_3_ thin films deposited on different oriented substrates were further studied for surface topography. The RMS values of *β*-Ga_2_O_3_ thin films on Si (100), Si (110), and Si (111) were reported as 2.54 nm, 2.47 nm, and 2.31 nm, respectively. It showed a decrement in the order of substrate Si (100) > Si (110) > Si (111).

#### 3.1.6. Based on Doping

Zhang et al. [100] fabricated a Si-doped *β*-Ga_2_O_3_-based SB-UVPD using the magnetron sputtering method. For the deposition of undoped and Si-doped *β*-Ga_2_O_3_ thin films, they used sputtering parameters of 100 W, 25 SCCM Ar gas flow, 0.5 Pa working pressure and 90 min deposition time. Furthermore, morphological characterization of the samples was performed using FESEM and AFM. They reported the Si-doped *β*-Ga_2_O_3_ sample film thickness of 950 nm and a grain size distribution with an average of 40 nm. Moreover, AFM analysis showed an island growth on the surface of both undoped and Si-doped *β*-Ga_2_O_3_ samples, and surface roughness was reported to be increased upon Si doping on *β*-Ga_2_O_3_. Similarly, Zhang et al. [101] reported morphological properties of Nb-doped *β*-Ga_2_O_3_-based solar-blind UV photodetector fabricated by RF magnetron sputtering. They found that there was no obvious change in the grain size of undoped *β*-Ga_2_O_3_ upon Nb doping, and an almost similar grain size of undoped *β*-Ga_2_O_3_ and Nb-doped *β*-Ga_2_O_3_ was observed.

### 3.2. Optical Properties

The optical characteristics of thin films, including transmittance, absorbance, and the energy bandgap (Eg), are crucial in determining the performance and effectiveness of photodetectors. The absorption coefficient of a material reflects its ability to absorb light at various wavelengths. A high absorption coefficient in the intended wavelength range ensures that most of the incident light is absorbed, resulting in a greater number of electron–hole pairs and a stronger electrical output. Materials with higher absorption coefficients are used for photodetectors because they offer greater sensitivity and efficiency. A semiconductor material’s E_g_ determines the wavelength range of light to which the photodetector may respond. Only photons with energy equal to or greater than E_g_ can excite electrons from the valence to conduction bands, resulting in an electrical signal. Some of the literature is discussed below.

#### 3.2.1. Based on Annealing Temperature

**Figure 18:**
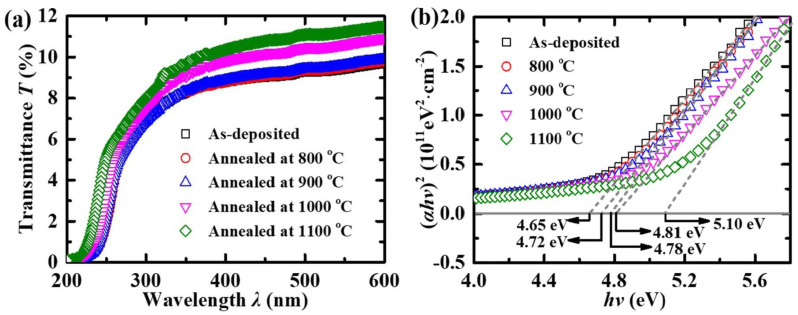
(**a**) Transmittance vs. wavelength curve and (**b**) Tauc plot of the as-deposited Ga_2_O_3_ sample and annealed samples. Adapted from ref. [95]. CC by 4.0.

Hu et al. [95] reported the influence of annealing temperature on the optical transparency and E_g_ of *β*-Ga_2_O_3_-based SB-UVPD, using post-annealing temperatures of 800 °C, 900 °C, 1000 °C, and 1100 °C, respectively. Figure 18 shows the optical transmittance and Tauc plot for E_g_. The annealed sample showed a blue shift. E_g_ of as-deposited Ga_2_O_3_ was 4.65 eV, and upon post-annealing, the energy bandgap increases from 4.67 eV to 5.13 eV for 800 °C, 900 °C, 1000 °C, and 1100 °C annealed samples (*β*-Ga_2_O_3_). They found that the post-annealing temperature increased the energy bandgap of the as-deposited Ga_2_O_3_ thin film at higher temperatures.

Similarly, li et al. [96] reported on the optical transmittance and energy bandgap of as-deposited Sn-doped Ga_2_O_3_ and annealed Sn-doped Ga_2_O_3_ (900 °C) in nitrogen and air atmospheres. According to the optical analysis, the thin film’s transmittance sharply decreased, attributed to absorption in the solar-blind region, as reported in the articles. The maximum transmittance at the wavelength was 73%. Moreover, the energy bandgap was determined from a Tauc plot. The energy bandgap of as-deposited Sn-doped Ga_2_O_3_ was reported as 4.86 eV, while that annealed in a nitrogen atmosphere was reported as 4.94 eV and that in an air atmosphere was 4.89 eV.

#### 3.2.2. Based on Growth Temperature

Cui et al. [97] reported an optical energy bandgap for *β*-Ga_2_O_3_-based SB-UVPD fabricated by PVD magnetron sputtering. A *β*-Ga_2_O_3_ thin film was deposited on the substrate at growth temperatures ranging from 450 °C to 700 °C. Furthermore, the E_g_ of each sample was determined using a Tauc plot. The obtained energy bandgaps of samples grown at 450 °C, 500 °C, 550 °C, 600 °C, 650 °C, and 700 °C were reported as 4.75 eV, 4.80 eV, 4.83 eV, 4.85 eV, 4.90 eV, and 4.94 eV, respectively. The results indicate that the energy bandgap increased with increasing growth temperature. Similarly, Wang et al. [102] reported the *β*-Ga_2_O_3_-based SB-UVPD’s optical energy bandgap, fabricated by the PVD magnetron sputtering method at growth temperatures of 550 °C, 650 °C, and 750 °C, respectively. The sputtering power was set to 70 W, and an argon gas flow rate of 25 SCCM was used with a base pressure of 1 × 10^−4^ Pa. Furthermore, the optical energy bandgap of *β*-Ga_2_O_3_ thin films grown at 550 °C, 650 °C, and 750 °C, determined from Tauc plots, was reported as 4.95 eV, 4.97 eV, and 4.99 eV, respectively. The results show that as growth temperature increases, the optical bandgap tends to increase.

#### 3.2.3. Based on Sputtering Power

Wang et al. [77] reported on the optical properties, such as transmittance and energy bandgap, of *β*-Ga_2_O_3_-based SB-UVPD fabricated at different sputtering powers, ranging from low to high values. They found that all *β*-Ga_2_O_3_ thin films fabricated had transmittance of over 95% in the UV–Visible wavelength region. Moreover, they determined the energy bandgap using the Tauc plot. They reported band energy gaps of 5.27 eV, 5.07 eV, and 4.90 eV for samples prepared at sputtering powers of 60 W, 100 W, and 150 W, respectively. They found that the energy bandgap at lower sputtering power (60 W) was more than at higher sputtering power (150), which might be due to diffusion of Al impurities of the sapphire substrate (α-Al_2_O_3_ substrate) on the thin film [77].

#### 3.2.4. Based on the Thickness of Films

Kaur et al. [39] reported on *β*-Ga_2_O_3_-based SB-UVPD with different thicknesses, i.e., 5 nm, 20 nm, 50 nm, 100 nm, and 300 nm, respectively, and studied the impact on the optical properties. Upon analysis of the optical result, they reported that all samples have an optical transmission of 80% at higher wavelengths and absorb energy in the UV-C region. Moreover, the optical bandgap of the samples decreased with increasing *β*-Ga_2_O_3_ thin-film thickness.

#### 3.2.5. Based on the Doping of the Element

Guo et al. [103] reported an intrinsic *β*-Ga_2_O_3_ and Zn-doped *β*-Ga_2_O_3_-based SB-UVPD using the PVD sputtering method. They varied the Zn doping concentration, i.e., 0.69, 0.88, 0.96, 1.83, 2.49, and 3.03, on intrinsic *β*-Ga_2_O_3_. They reported that Zn doping of *β*-Ga_2_O_3_ decreased the intrinsic *β*-Ga_2_O_3_ energy bandgap (Eg). The energy bandgap of intrinsic *β*-Ga_2_O_3_ was estimated using the Tauc plot as 4.92 eV, which decreased to 4.7 eV when Zn 3.03 atom% was doped with *β*-Ga_2_O_3_. Similarly, Zhang et al. [101] reported on a Nb-doped *β*-Ga_2_O_3_-based SB-UVPD fabricated by PVD magnetron sputtering, using 80 W for *β*-Ga_2_O_3_ and 10 W for Nb. The optical energy bandgaps of intrinsic *β*-Ga_2_O_3_ and Nb-doped *β*-Ga_2_O_3_ (0.3 atom%) were determined using a Tauc plot, reported as 5.04 eV and 4.93 eV, respectively. The optical energy bandgap results indicate a decrease in the bandgap when Nb is doped into intrinsic *β*-Ga_2_O_3_.

#### 3.2.6. Based on Gas Flow: Argon/Oxygen Ratio

Bhowmick et al. [104] fabricated *β*-Ga_2_O_3_-based SB-UVPD using the PVD magnetron sputtering method by varying the Ar to O_2_ ratio variation to 1:0, 1:1, and 1:2. Further, the energy bandgap of the samples was reported, which was measured by the Tauc plot. The energy bandgap of the samples fabricated at Ar:O_2_ ratios of 1:0, 1:1, and 1:2 was reported as 4.17 eV, 4.20 eV, and 4.15 eV, respectively. The energy bandgap decreased with increasing oxygen concentration, possibly due to thin-film degradation at higher oxygen levels.

### 3.3. Performance Characteristics of SB-UVPD (Electrical Properties)

#### 3.3.1. Based on Post-Annealing Temperature

**Figure 19:**
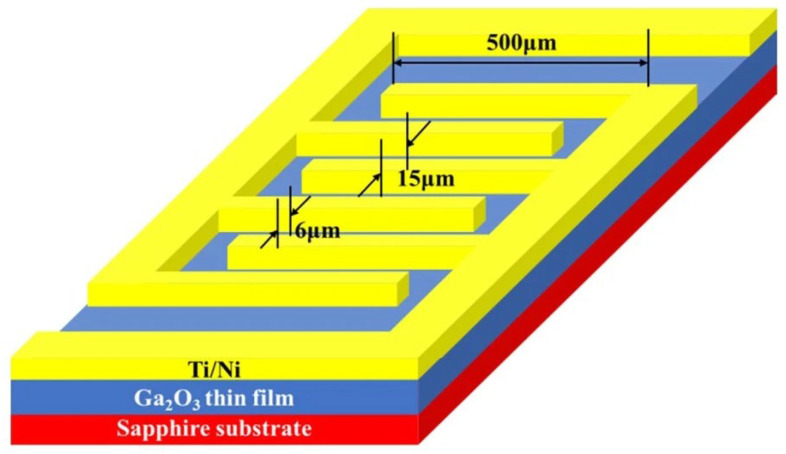
Pictorial presentation of *β*-Ga_2_O_3_-based SB-UVPD on sapphire substrate. Adapted from Ref. no. [95]. CC by 4.0.

Hu et al. [95] reported on the performance characteristics of a *β*-Ga_2_O_3_-based SB-UVPD based on as-deposited and annealed (800 °C, 900 °C, 1000 °C, and 1100 °C) thin films, as shown in Figure 19.

**Figure 20:**
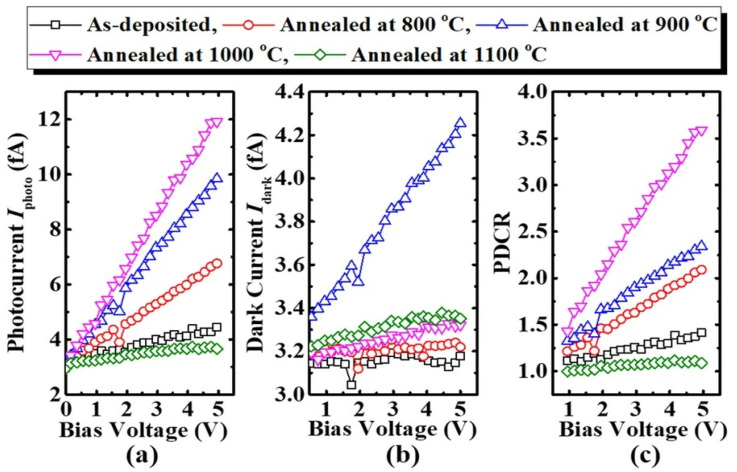
(**a**) Photocurrent (I_photo_), (**b**) dark current (I_dark_), and (**c**) PDCR of as-deposited and annealed (800 °C, 900 °C, 1000 °C, and 1100 °C) *β*-Ga_2_O_3_-based SB-UVPD. Adapted from Ref. no. [95]. CC by 4.0.

**Figure 21:**
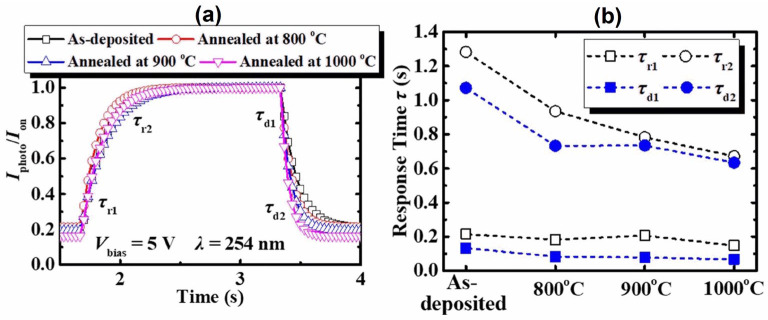
(**a**) Time-dependent photo response data and (**b**) rise and decay time of as-deposited and annealed (800 °C, 900 °C, and 1000 °C) *β*-Ga_2_O_3_-based SB-UVPD. Adapted from Ref. no. [95]. CC by 4.0.

To measure the performance characteristics of the devices, a bias voltage of ±5 V, illumination light with a 254 nm wavelength, and a light intensity of 0.5 mW/cm^2^ were used. They reported that the device fabricated at a higher annealing temperature (up to 1000 °C) exhibited a larger photocurrent, which increased with increasing annealing temperature, as indicated in Figure 20. However, a photodetector fabricated at an annealing temperature of 1100 °C produced a photocurrent that was lower than that of an as-deposited Ga_2_O_3_-based device. Moreover, it was reported that the response time was decreased for the annealed-based photodetector. The response time of the as-deposited Ga_2_O_3_-based photodetector was reported to be 0.215 s, whereas for the photodetector annealed at a higher temperature (1100 °C), it was observed to be 0.148 s, as shown in Figure 21. The decay time for the as-deposited Ga_2_O_3_-based photodetector was 0.133 s, which was reduced to 0.067 s after annealing at 1100 °C.

Similarly, Wang et al. [94] reported on a *β*-Ga_2_O_3_-based SB-UVPD by varying different post-annealing temperatures (500 °C, 700 °C, and 900 °C). To measure the performance characteristics, 254 nm and 365 nm illumination were used with a bias voltage of 10 V. The dark current of as-deposited Ga_2_O_3_ samples was reported as 1.81 × 10^4^ nA, while for post-annealed-based devices at 500 °C, 700 °C, and 900 °C, respectively, this was 4.37 nA, 0.73 nA, and 0.46 nA. The dark current decreased as the annealing temperature increased. Moreover, the PDCR of the as-deposited Ga_2_O_3_ sample was observed to be 4.61, whereas for the samples annealed at 500 °C, 700 °C, and 900 °C, the values reported were 1.56 × 10^3^, 1.27 × 10^2^, and 1.42 × 10^2^, respectively, under light illumination of 254 nm wavelength. The responsivity and detectivity of as-deposited Ga_2_O_3_ and annealed devices at 500 °C, 70 °C, and 900 °C were reported as 5.62, 0.45, 0.0061, and 0.0043, and 4.04 × 10^11^, 2.11 × 10^12^, 6.91 × 10^10^, and 6.11 × 10^10^, respectively. And lastly, response times (rise/decay times) for as-deposited Ga_2_O_3_ and annealed samples at 500 °C, 700 °C, and 900 °C were reported as 2.68 s/5.45 s, 1.72 s/0.42 s, 1.57 s/0.17 s, and 1.03 s/0.13 s, respectively. Zhou et al. [57] fabricated a *β*-Ga_2_O_3_ thin film-based SB-UVPD via the PVD magnetron sputtering method. A *β*-Ga_2_O_3_ thin film was deposited at 150 W, with an argon flow rate of 40 SCCM for 1.5 h, and then post-annealed at 900 °C. For MSM structure device fabrication, a Ti/Au electrode finger with a spacing of 10 μm was fabricated on the sample using magnetron sputtering. Fabricated photodetector I-V characteristics were measured under 254 nm UV illumination at an incident power density of 500 μW/cm^2^. The dark current of the photodetector was reported to be 82 fA at a 10 V bias. Furthermore, when the photodetector was exposed to 254 nm UV light illumination, the photocurrent was reported to be 29 nA. The PDCR, responsivity, and detectivity were obtained as 3.58 × 10^5^, 1.93 AokW^−1^, and 6.53 × 10^13^ Jones, respectively. And Jiao et al. [105] fabricated a *β*-Ga_2_O_3_ thin film-based SB-UVPD using PVD magnetron sputtering with post-annealing temperatures ranging from 500 °C to 900 °C. The performance characteristics of the amorphous and annealed Ga_2_O_3_ thin-film-based solar-blind UV photodetector were analyzed using 254 nm and 365 nm UV illumination. The I-V measurements were conducted for both amorphous and annealed Ga_2_O_3_ thin-film-based solar-blind UV photodetectors at a 20 V bias. The dark current was reported as 0.1 pA for amorphous and 1.1 pA under 365 nm UV illumination. Furthermore, at 254 nm UV light illumination, the photocurrent was reported to jump to 0.1 nA. Responsivity was measured at 122.7 μA/W. Moreover, I-V characteristics of annealed Ga_2_O_3_ thin film-based photodetector were measured, and the dark current of annealed samples at 500 °C was observed as 0.02 nA at a bias voltage of 30 V. Increasing the annealing temperature from 500 °C to 900 °C, the dark current was reported as 1 pA. The photocurrent at 254 nm, following UV light illumination of all annealed samples, was reported to be the same as the dark current; no obvious change was observed.

#### 3.3.2. Based on Growth Temperature

**Figure 22:**
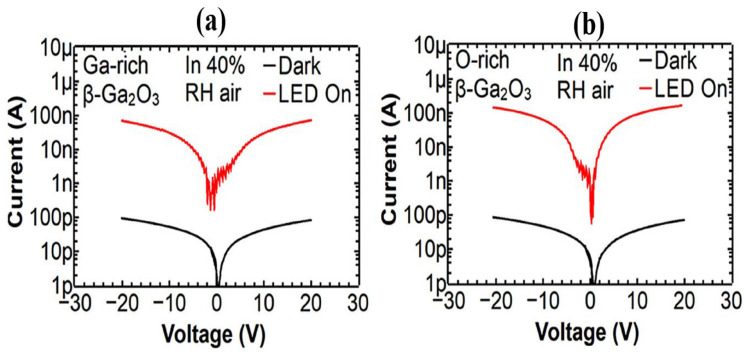
I-V characteristic of (**a**) Ga-rich and (**b**) O-rich fabricated SB-UVPD (in 40% RH). Adapted from Ref. no. [107]. CC by 4.0.

Peng et al. [106] fabricated a *β*-Ga_2_O_3_ thin film-based SB-UVPD using the PVD magnetron sputtering method at a growth temperature of 750 °C. For the deposition of *β*-Ga_2_O_3_ thin films, sputtering parameters included a plasma power of 80 W, a base chamber pressure of 10^−7^ mbar, and a growth temperature of 750 °C. For the fabrication of the photodetector electrode in the Ti/Au MSM structure, a pattern was generated using lithography on the samples. For the analysis of the performance characteristics of a *β*-Ga_2_O_3_ thin film-based solar-blind UV photodetector, 254 nm and 365 nm UV light illumination with an incident power density of 200 μW/cm^2^ were utilized. The dark current of the photodetector was reported as 10 pA at 5 V biassing voltage. I-V current analysis under 365 nm UV illumination does not show any sensitivity. In contrast, under 254 nm UV illumination, the photodetector showed improved sensitivity, with a photocurrent of 1.15 µA. The PCDR was 10^5^, and at a 10 V bias, the photoresponsivity was reported as 0.89 A/W. Similarly, Wang et al. [94] fabricated a *β*-Ga_2_O_3_ SB-UVPD by PVD magnetron sputtering at an optimal growth temperature of 550 °C, 650 °C, and 750 °C. The MSM structure photodetector electrode was fabricated using a Ti/Au layer with three pairs of fingers. Furthermore, UV illumination at 254 nm and 365 nm was used to analyze the performance characteristics. Furthermore, the I-V characteristics of the fabricated photodetector were measured; the dark current was reported to be ~0.1 nA, and no sensitivity was observed upon illumination with 365 nm wavelength UV light. However, under 254 nm solar-blind UV light illumination and an incident power density of 0.3 mW/cm^2^, the photocurrent was reported to be 1 μA at a 5 V biassing voltage. The sensitivity of the blind photodetector was reported to be excellent. Lui et al. [20] also reported on the performance characteristics of a *β*-Ga_2_O_3_ thin-film-based SB-UVPD fabricated by PVD magnetron sputtering, with a substrate temperature of 750 °C. For the deposition of the *β*-Ga_2_O_3_ thin film, they used the following sputtering parameters: power, 70 W; argon gas flow, 24.8 SCCM; base vacuum chamber pressure, 2.4 × 10^−4^ Pa; and substrate temperature, 750 °C. A Ti/*β*-Ga_2_O_3_/Ni Schottky junction-based photodetector was fabricated, and its performance characteristics were analyzed. The I-V measurement was performed using 254 nm UV illumination at a 10 V bias and an incident light density of 247.8 μW/cm^2^. The dark current was reported as low (13.2 pA), while the photocurrent was reported as 4.58 µA. The PDCR, responsivity, EQE, and specific detectivity were reported as 2.83 × 10^5^, 144.46 A·W^−1^, 64.711%, and 7.29 × 10^14^ cm. Hz^1/2^·W^−1^ (Jones), respectively. Chen et al. [59] reported a *β*-Ga_2_O_3_ thin film-based SB-UVPD fabricated by PVD magnetron sputtering method at different growth temperatures of 600 °C, 650 °C, and 700 °C, with a deposition time of 1.5 h at 100 W. For the fabrication of an MSM structure-based photodetector, a platinum (Pt) electrode was deposited by magnetron sputtering on the surface of the optimal-condition samples (700 °C) using a mask. Furthermore, performance characteristics were analyzed under 254 nm UV illumination at an incident power density of 70 μW/cm^2^ with a 10 V biassing voltage. According to the I-V characteristics, the photodetector’s dark current was reported as 3.5 pA. When 254 nm UV light was incident on the photodetector surface, the photocurrent was measured at 42 nA. The PDCR was found to be over 10^4^. Moreover, photoresponsivity and response time were reported as 30 mA/W and rise time (0.07 s/0.53 s) and decay time (0.06 s/0.16 s), respectively. And, Sharma et al. [107] reported on a *β*-Ga_2_O_3_ thin film-based SB-UVPD fabricated using the PVD magnetron sputtering method at a growth temperature of 800 °C and investigated the performance characteristics at room temperature using air humidity of 40% or 90%. I-V characteristics were performed over a bias range of −20 to +20 V under 254 nm UV-C illumination with an incident power density of 0.09 mW cm^−2^. Figure 22 shows the I-V characteristics of the Ga- and O-rich fabricated device in 40% relative humidity (RH). The devices exhibited a photo-to-dark current ratio of ~10^3^, along with a responsivity of 2 A/W and a detectivity of 1.2 × 10^13^ Jones. Operation at ~90% relative humidity yielded the fastest UV-C response but increased dark current due to humidity-induced surface adsorption, reducing band bending and enhancing carrier conduction.

#### 3.3.3. Based on Doping Element

**Figure 23:**
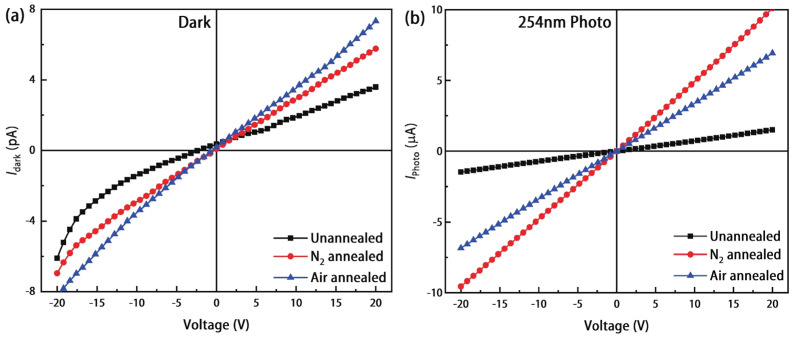
I-V curve of (**a**) dark current and (**b**) photocurrent of Sn-doped *β*-Ga_2_O_3_-based SB-UVPD at different conditions. Adapted with permission from ref. [96]. Copyright © 2024. IOP Publishing.

Li et al. [96] reported on a Sn-doped Ga_2_O_3_-based SB-UVPD and analyzed the performance characteristics under illumination of 254 nm using a bias voltage of ±20 V. Upon analyzing the device performance characteristics, the PDCR of the as-deposited Sn-doped Ga_2_O_3_ and annealed Sn-doped *β*-Ga_2_O_3_ samples at nitrogen and air atmospheres at 900 °C were reported to be 10^5^ and 10^6^, respectively. The dark current and photocurrent of as-deposited Sn-doped Ga_2_O_3_ were reported to be the same (10^5^ range), as indicated in Figure 23. Moreover, dark current was observed to be increased for annealed samples compared with as-deposited Sn-doped Ga_2_O_3_. At 254 nm wavelength illumination, the maximum photocurrent was generated for all samples.

Similarly, Qian et al. [108] reported on a Mg-doped *β*-Ga_2_O_3_ thin film-based SB-UVPD fabricated by the PVD magnetron sputtering method. Before fabricating the photodetector, they deposited Mg-doped *β*-Ga_2_O_3_ thin films with varying Mg concentrations of 4.92, 6.88, and 8.58 atom% in *β*-Ga_2_O_3_ and subsequently annealed them at 600 °C, 700 °C, and 800 °C, respectively. After investigating all results, including XRD, SEM/EDX, and XPS analysis, the Mg-doped *β*-Ga_2_O_3_ (4.92 atom%) at 800 °C was the best among all samples. Furthermore, a photodetector with optimal Mg (4.92 atom%) doping in *β*-Ga_2_O_3_ was fabricated. For I-V characteristics, UV light illumination at 254 nm and 365 nm was conducted with a 10 V bias voltage. The dark current of a Mg 4.92 atom (%)-doped *β*-Ga_2_O_3_ photodetector was reported as 4.1 pA. The photo current of the device under 365 nm UV illumination showed no significant change, with a value of 6.2 pA. Moreover, a photocurrent of 35.6 nA at a 10 V bias voltage for the photodetector, illuminated with 254 nm UV light and doped with Mg 4.92 atoms (%) in *β*-Ga_2_O_3_, was reported to show a significant jump, implying that the photodetector was very sensitive to 254 nm UV light illumination. The photosensitivity, responsivity, and decay time were reported as 8.7 × 10^5^%, 23.8 mA/W, and 0.02 s. Zhang et al. [101] reported on an Nb-doped *β*-Ga_2_O_3_-based SB-UVPD using the PVD magnetron sputtering method. To measure the performance characteristics of the device, an I-V measurement was conducted using 254 nm UV light illumination with an incident optical power of 100 μW/cm^2^. The dark current of the fabricated device named #2 was reported as 0.2 nA. When UV light of 254 nm illumination falls on the device, the photocurrent is reported as 50 nA. Moreover, the sensitivity of the device was recorded as higher than 100, and response rise time and decay time were recorded as (0.524 s/2.83 s) and 0.095 s, respectively. Guo et al. [103] fabricated an intrinsic *β*-Ga_2_O_3_ and Zn-doped *β*-Ga_2_O_3_-based SB-UVPD using the PVD sputtering method. The Zn concentration of 0.69, 0.88, 0.96, 1.83, 2.49 and 3.03 atom% was doped with *β*-Ga_2_O_3_ thin films. Furthermore, to measure the solar-blind photodetector performance of the thin films, a Zn 3.03% atom-doped *β*-Ga_2_O_3_-based MSM device was fabricated. For measuring the I-V characteristics, 254 nm UV light illumination was selected, with an intensity of 15 μW/cm^2^. The dark current of an intrinsic *β*-Ga_2_O_3_-based photodetector was reported as 0.43 nA, and at 254 nm light illumination, the photo current obtained was 13 nA at 10 V biassing. For the Zn-doped *β*-Ga_2_O_3_ photodetector, the dark current was reported to be 0.31 nA, and under 254 nm UV illumination, a photocurrent of 34 nA was recorded. Further, the ratio of photo-to-dark current for intrinsic *β*-Ga_2_O_3_ was reported to be 30, while for Zn-doped *β*-Ga_2_O_3_, as 110. The rise time and decay time for the intrinsic *β*-Ga_2_O_3_ MSM photodetector were reported as (3.39 s and 20.30 s) and (0.60 s and 0.05 s). On the other hand, the rise and decay times of Zn-doped *β*-Ga_2_O_3_ MSM photodetector are reported as (1.95 s, 15.04 s) and 0.25 s, respectively. And Zhang et al. [100] reported on a Si-doped *β*-Ga_2_O_3_ SB-UVPD fabricated by the magnetron sputtering method. Upon analysis, they reported that incorporating Si into *β*-Ga_2_O_3_ thin films significantly improves solar-blind photodetector performance. Under an applied bias of 15 V, the Si-doped *β*-Ga_2_O_3_ device exhibits an ultralow dark current of 32.2 pA and a markedly enhanced light-to-dark current ratio of 3.2 × 10^4^ at an incident optical power density of 2.4 μW cm^−2^ at UV light illumination of 254 nm wavelength. Moreover, responsivity of 1.146 A W^−1^, a specific detectivity of 7.14 × 10^11^ Jones, and an EQE of 5.6% were reported, representing enhancements of more than one order of magnitude relative to the undoped device. Moreover, the rise and decay times were reported as (3.23 s/1.55 s)/(1.62 s/0.31 s).

#### 3.3.4. Based on Substrate Type

Yu et al. [58] fabricated a *β*-Ga_2_O_3_ thin-film-based SB-UVPD using the PVD magnetron sputtering on different substrates, including MgO (100), MgAl_2_O_4_ (100) (referred to as MAO), and α-Al_2_O_3_ (0001). To measure the PD’s performance characteristics, I-V measurements were conducted. In I-V characteristics measurements, 254 nm and 365 nm UV illumination were used, with a light density of 300 μW/cm^2^ at a 5 V bias. The dark current for all samples was reported as 0.2 pA, and no photocurrent was observed when the samples were illuminated with 365 nm UV light. Furthermore, at 254 nm UV light illumination, a *β*-Ga_2_O_3_-based SB-UVPD deposited on MgO, MAO, and α-Al_2_O_3_ substrate reported photocurrent of 0.95 µA, 0.56 µA, and 71 nA, respectively. Moreover, the PDCR ratio was reported to be the highest for the MgO substrate compared to other PDs (>2 × 10^4^). Also, *β*-Ga_2_O_3_ deposited on MgO photodetector showed the best results, such as higher responsivity of 0.1 A·W^−1^, detectivity of 4.3 × 10^12^ Jones, and EQE of 0.49 under 254 nm UV light illumination, compared to other devices.

#### 3.3.5. Based on Substrate Orientation

Kaur et al. [85] reported on a *β*-Ga_2_O_3_-based SB-UVPD fabricated by RF magnetron sputtering on Si (100), (110), and (111) substrates. For fabricating a photodetector, a Cr/Au interdigitated electrode was deposited on the surface by thermal evaporation using a shadow mask. Furthermore, the photodetector’s performance characteristics were analyzed using 254 nm UV illumination at a 5 V bias voltage. I-V characteristics were measured for all three samples; the Si (111) oriented SB-UVPD reported the highest dark current (~7 × 10^−8^ A). The PDCR values reported for Si (100), Si (110) and Si (111) oriented photodetectors were ~5, ~9, and ~54, respectively. Moreover, the highest responsivity was reported for Si (111) at 0.6 A/W, while the best optimal response times, i.e., the rise and decay times of the Si (100)-oriented photodetector, were reported as 0.003 s and 0.012 s, respectively.

#### 3.3.6. Based on Film Thickness

Kaur et al. [39] reported on a *β*-Ga_2_O_3_-based SB-UVPD, fabricated by RF magnetron sputtering. For fabricating *β*-Ga_2_O_3_ thin films, sputtering parameters included a power of 100 W, a base vacuum pressure of 8 × 10^−7^ Torr, and a deposition temperature of 500 °C. Various thicknesses, including 5 nm, 20 nm, 50 nm, 100 nm, and 300 nm, were fabricated using *β*-Ga_2_O_3_ SB-UVPD. Furthermore, device performance characteristics were analyzed under light illumination at 254 and 365 nm. All fabricated *β*-Ga_2_O_3_-based photodetectors of various thicknesses (5 nm, 20 nm, 50 nm, 100 nm, and 300 nm) reported solar blindness for 365 nm illumination light but generated a photocurrent when a light illumination of 254 nm fell on the surface of the samples. The response time was reported to improve as *β*-Ga_2_O_3_ thickness increased, i.e., as the thickness was increased from 5 to 300 nm, while the rise and fall times were reported to decrease from 8.37 s to 287 ms and 7.61 s to 178 ms, respectively.

In addition to the detailed review of the RF-sputtered *β*-Ga_2_O_3_-based SB-UVPD, as discussed above, it is important to assess device performance from a comparative perspective. In this regard, Table 3 presents a comprehensive comparison of RF-sputtered *β*-Ga_2_O_3_-based solar-blind UV photodetector performance metrics (dark current, photocurrent, PDCR, responsivity, detectivity, and rise/decay time) deposited on different substrates.

## 4. Challenges Associated with RF-Sputtered *β*-Ga_2_O_3_-Based Solar-Blind UV Photodetector

From the above discussion, RF magnetron sputtering is attractive for *β*-Ga_2_O_3_ SB-UVPDs due to its scalability and process compatibility; however, device performance is frequently limited by growth-induced non-idealities such as crystallinity, morphologies, and temperature control, particularly for sputtered *β*-Ga_2_O_3_ on heterogeneous substrates. The following major concerns should be addressed before commercializing this method.

### 4.1. Stoichiometric β-Phase Formation and Phase/Transition Control

A central limitation of RF sputtering is that as-deposited Ga_2_O_3_ thin films can be amorphous or weakly crystalline and obtaining device-grade *β*-Ga_2_O_3_ often requires either elevated deposition temperature and/or post-annealing. For example, systematic RF-sputtering studies show that *β*-Ga_2_O_3_ signatures emerge only beyond a threshold deposition temperature, while XRD can still indicate poor crystallinity in as-deposited layers; post-annealing improves crystallinity but yields polycrystalline films and may also introduce secondary phases such as γ-Ga_2_O_3_ in some cases [109]. Likewise, RF-sputtered amorphous Ga_2_O_3_ films have been demonstrated to crystallize into monoclinic *β*-Ga_2_O_3_ after high-temperature oxygen annealing (e.g., 1000 °C), but the resulting microstructure and optical properties remain sensitive to the oxygen partial pressure during deposition, indicating that phase formation and stoichiometry are tightly coupled to reactive sputtering conditions. This requirement is particularly relevant for SB-UVPDs because the β-phase, crystallographic texture, and defect chemistry directly determine the sharpness of the absorption edge, carrier transport, and leakage pathways [11,110].

Implication for SB-UVPDs: A key practical bottleneck is therefore the need to realize stoichiometric, phase-pure *β*-Ga_2_O_3_ while keeping the thermal budget compatible with target substrates and backend processing. The challenge is not merely “crystallization”, but reproducible β-phase stabilization without introducing stress-related microcracking or parasitic phases, which can occur depending on oxygen partial pressure and post-anneal conditions [110].

### 4.2. Oxygen Vacancies: Conductivity Tuning Versus Dark Current and PPC

Among sputtered *β*-Ga_2_O_3_ films on foreign substrates, oxygen vacancies and oxygen-related deep states are widely recognized as dominant defects influencing electrical transport and photoresponse. Oxygen-vacancy-related traps can elevate dark current and degrade mobility, and they are strongly linked to persistent photoconductivity (PPC), which slows detector recovery and undermines high-speed operation [111].

A representative quantitative study on sputtered *β*-Ga_2_O_3_ SBPDs shows that oxygen-plasma treatment can significantly reduce vacancy-related effects and PPC, leading to a dark current reduction by ~one order of magnitude and a marked acceleration of response time (e.g., from seconds to sub-seconds), thereby explicitly demonstrating the defect-engineering leverage available for sputtered films. In parallel, vacancy modulation studies (even when using non-sputtered growth) reinforce the general physical trend that lower oxygen-vacancy content correlates with lower dark current and improved signal-to-noise metrics, underscoring oxygen-vacancy control as a universal handle for Ga_2_O_3_ photodetectors [110,111].

Implication for SB-UVPDs: The key challenge is that oxygen vacancies in sputtered *β*-Ga_2_O_3_ are not “purely detrimental”; they can also contribute to photoconductive gain via trapping/release dynamics. Consequently, the device optimization space is inherently multi-objective: minimize vacancy-driven leakage and PPC while retaining sufficient carrier generation/collection and avoiding over-compensation that suppresses photoresponse [111].

### 4.3. Crystallinity–Dark Current Trade-Off in RF-Sputtered β-Ga_2_O_3_

For RF-sputtered *β*-Ga_2_O_3_ SB-UVPDs, improving crystallinity by increasing deposition temperature or employing aggressive post-annealing can enhance structural ordering and potentially improve carrier transport. However, in hetero-integrated sputtered films, crystallization and defect evolution are intertwined: lattice-mismatch-induced distortions and oxygen-deficient regions can persist and act as trap centres, which, in turn, raise dark current and promote PPC [109,110,111]. This establishes a device-relevant trade-off that is particularly important for sputtered *β*-Ga_2_O_3_ SB-UVPDs: the conditions that improve crystallinity (temperature/anneal/oxygen chemistry) must be tuned simultaneously to avoid defect-assisted leakage paths and unfavourable trap distributions that penalize PDCR and detectivity [110,111].

Moreover, rather than treating “crystallinity” as an isolated goal, sputtered *β*-Ga_2_O_3_ SB-UVPD optimization should target defect-controlled crystallinity, i.e., crystalline *β*-Ga_2_O_3_ with a defect population engineered to suppress leakage and PPC while maintaining high photoresponse [111].

### 4.4. Reproducible Schottky Contact Formation on Sputtered β-Ga_2_O_3_

A major performance limiter in SB-UVPDs is the stability and reproducibility of the metal/*β*-Ga_2_O_3_ interface, because Schottky barrier height (SBH), barrier homogeneity, and interface traps directly control dark current, barrier-limited leakage, PDCR, and detectivity. Electrical characterization across multiple metals on *β*-Ga_2_O_3_ shows that measured SBHs can vary widely with metal choice and measurement method, and that certain contacts (e.g., Au, Pd) can exhibit spatially inhomogeneous barriers, associated with interfacial reactions/alloy formation and non-ideal transport [109]. For RF-sputtered *β*-Ga_2_O_3_, this issue can be exacerbated because sputtered surfaces may exhibit higher roughness, higher defect density, and more complex surface states than high-quality epitaxial counterparts, making SBH and contact leakage more sensitive to surface preparation, post-metallization anneals, and passivation schemes. The effectiveness of plasma-based processing in suppressing vacancy-related traps and PPC further suggests that surface/interface conditioning is a critical lever for achieving reproducible Schottky interfaces in sputtered SB-UVPDs [109,111].

Implication for benchmarking: Because contact quality can dominate device metrics, fair comparisons across reports require explicit reporting of the metal stack, surface cleaning, post-metallization anneals, and contact geometry, alongside film growth/anneal parameters [109].

### 4.5. Process Window Sensitivity and Large-Area Reproducibility

Although RF sputtering is intrinsically scalable, Ga_2_O_3_ reactive sputtering is sensitive to oxygen chemistry and plasma conditions; for example, oxygen flow/DC target potential can influence composition and oxygen content, which, in turn, affect refractive index and film quality. Similarly, sputtering gas composition, substrate temperature, and post-annealing temperature have been shown to modify crystalline signatures and optical properties of sputter-deposited Ga_2_O_3_ [109,112].

Key commercialization-relevant challenge: Achieving device-to-device uniformity in dark current and response speed requires tight control over stoichiometry and defect density, not just thickness and texture. Standardizing reporting of illumination conditions (wavelength, intensity, spot size), biassing, effective area, and time-constant definitions is also necessary to enable robust cross-lab comparisons and meaningful benchmarking [11,111].

## 5. Future Scope of PVD RF-Sputtered *β*-Ga_2_O_3_-Based SB-UVPDs for Next-Generation Optoelectronics

*β*-Ga_2_O_3_ is an emerging wide-bandgap semiconductor material for SB-UVPD due to its ultra-wide bandgap, high breakdown field, thermal robustness, and excellent chemical stability in harsh environments, as discussed in the introductory sections. In the coming decade, photodetectors fabricated using PVD RF sputtering are expected to transition from laboratory prototypes to scalable, low-cost, and application-ready next-generation optoelectronic devices. One key future development will be precision-controlled thin-film engineering. Advances in RF sputtering, such as real-time plasma diagnostics, adaptive power modulation, and multi-target co-sputtering, will allow researchers to finely tune oxygen vacancy concentration, grain orientation, and interface quality. These improvements will directly enhance device metrics, including responsivity, dark current suppression, and temporal response. The ability to deposit uniform *β*-Ga_2_O_3_ films on large-area wafers and flexible substrates will open new markets, including aircraft skins, space platforms, and environmental monitoring. Another significant trend will be the emergence of heterostructure-engineered PDs. By integrating sputtered *β*-Ga_2_O_3_ with materials such as AlN and AlGaO, designers will develop band-engineered architectures capable of ultrafast detection, high gain, and enhanced spectral selectivity. The PVD RF sputtering method will play a crucial role because it enables low-temperature deposition, which is compatible with CMOS platforms. As a result, fully integrated Ga_2_O_3_-based UV photodetector chips may become standard components in next-generation autonomous systems. Moreover, device miniaturization and nano-structuring enabled by lithography-assisted sputtering will lead to nano-ridge and nanocolumn photodetectors with enhanced photon-trapping abilities. These structures have the potential to reduce power consumption and enable battery-free UV sensor nodes for the Internet of Things (IoTs). In practical applications, sputtered *β*-Ga_2_O_3_ detectors are expected to redefine fields such as secure space-to-ground communication, missile plume detection, biomedical sterilization monitoring, and environmental UV hazard mapping. Their reliability in extreme radiation conditions makes them ideal for deep-space missions and planetary exploration.

Overall, the future of PVD RF-sputtered *β*-Ga_2_O_3_-based solar-blind UV photodetectors is geared toward scalable manufacturing, multifunctional heterostructures, ultrafast response times, and integration into intelligent optoelectronic systems. This combination positions *β*-Ga_2_O_3_ as a cornerstone material for the next era of UV photonics.

## 6. Conclusions

The present review assesses recent progress and challenges in PVD RF magnetron-sputtered *β*-Ga_2_O_3_-based SB-UVPDs. Different photodetector configurations and key performance parameters of the PVD RF sputtering method are systematically explored together with the fundamental material characteristics such as structural, optical and electrical properties. Attention is given to RF magnetron sputtering, highlighting how deposition parameters such as RF power, working pressure, deposition duration, target-to-substrate spacing, and substrate temperature strongly influence the crystallinity, microstructure, surface morphology, and optoelectronic properties of *β*-Ga_2_O_3_ thin films for SB-UVPDs. The review further discusses the role of post-deposition treatments, including thermal annealing and elemental doping, in regulating defect states, carrier transport, and overall SB-UVPDs performance characteristics. The electrical characteristics of *β*-Ga_2_O_3_-based SB-UVPDs are comparatively analyzed, focusing on growth and processing conditions during RF sputtering, thereby providing insight into structure–property–performance relationships. Despite its technological appeal as a scalable and cost-efficient deposition technique, RF magnetron sputtering faces fundamental limitations when applied to the fabrication of *β*-Ga_2_O_3_ SB-UVPDs. A primary difficulty lies in the fact that Ga_2_O_3_ thin films grown by sputtering are often structurally disordered at low temperatures, and reliable formation of device-quality β-phase material usually requires high substrate temperatures or subsequent thermal treatments. Such thermal requirements can conflict with substrate compatibility and may lead to stress accumulation, grain boundary, or unintentional phase transitions. At the same time, oxygen-related point defects, which are inherently sensitive to reactive sputtering conditions, play a crucial role in determining charge transport and thus in device performance. While these microstructural defects can enhance photoconductive gain through carrier trapping mechanisms, it raises leakage current levels and induces PPC, thereby compromising response speed and detector stability. Increasing crystallinity through thermal or oxygen-rich processing does not necessarily resolve these challenges, as defect redistribution and interface strain in heterogeneously integrated films can create additional leakage pathways that degrade detectivity and PDCR. Furthermore, device performance in these methods is influenced by the quality of metal/*β*-Ga_2_O_3_ junctions, where surface disorder and high trap densities in sputtered films often lead to non-uniform Schottky barriers and contact-dominated leakage currents. Finally, although sputtering is widely regarded as a large-area manufacturing technique, the narrow tolerance to variations in oxygen chemistry, plasma conditions, and thermal history presents a major obstacle to achieving wafer-level uniformity and reproducible device metrics. Collectively, these challenges indicate that the successful deployment of RF-sputtered *β*-Ga_2_O_3_ SB-UVPDs will require rigorous defect-aware process control and consistent benchmarking practices rather than improvements in crystallinity or scalability alone.

Overall, the present work promotes establishing RF magnetron sputtering as a reliable and scalable approach for fabricating high-quality *β*-Ga_2_O_3_-based SB-UVPDs, offering remarkable opportunities for their integration into next-generation high-performance optoelectronic and photonic systems.

## Figures and Tables

**Table 1:** Properties of the different polymorphs of gallium oxide [29,44].

Polymorphism	System	Space Group	Lattice Parameters
*α*	Hexagonal	R3*c*	*a* = 4.9825, *b* = 13.433
*β*	Monoclinic	C2/m	*a* = 12.214, *b* = 3.0371, *c* = 5.7981, *β* = 103.83
*γ*	Cubic	Fd3*m*	*a* = 8.22
*k*	Orthorhombic	Pna2_1_	*a* = 5.0463, *b* = 8.7020, *c* = 9.2833
*δ*	Cubic	Ia3	*a* = 9.491
*ɛ*	Hexagonal	P63mc	*a* = 2.9036, *a* = 9.2554

**Table 2:** Comparison of Ga_2_O_3_’s essential characteristics with other WBGs [29,44,45].

Properties	*β*-Ga_2_O_3_	GaN	SiC	Diamond	AlGaN	ZnO	ZnMgO
Energy bandgap (eV)	~4.6–4.9	~3.4	3.2(4H-SiC)	5.5	3.4–6.2	3.3	3.4–7.8
Breakdown field (MV/cm)	13	~3.3	3	10	3–5	3	3–5
Electron mobility (cm^2^/V-s)	300	1500	950	2200	1000 (for GaN)	200	100
Melting point (°C)	1795	1795	2830	Sublimations at ~3550	~1700	~1975	~1975
BFOM	3444	870	340	2000	870	500	700
Thermal stability	Yes	yes	yes	No	limited	yes	low

**Table 3:** Device performance comparison of some RF-sputtered *β*-Ga_2_O_3_-based SB-UVPDs.

Device	R (A/W)	I_dark_	PDCR	Detectivity D* (Jones)	Response Time (τ_r_/τ_d_) (s)	Ref.
Ga_2_O_3_/c-plane α-Al_2_O_3_ (0001)	-	82 fA	3.58 × 10^5^	6.53 × 10^13^	-	[57]
Ga_2_O_3_/α-Al_2_O_3_	0.89	10 pA	10^5^	-	-	[106]
Si-doped Ga_2_O_3_/α-Al_2_O_3_	1.146	32.2 pA	3.2 × 10^4^	7.14 × 10^11^	(3.23/1.55)/(1.62/0.31)	[100]
Zn (3.03%)-doped Ga_2_O_3_/α-Al_2_O_3_	-	0.31 nA	110	-	1.95/0.25	[103]
Ga_2_O_3_(500 °C, 700 °C, and 900 °C)/(0006) α-Al_2_O_3_	0.45, 0.0061, and 0.0043	4.37 nA, 0.73 nA, and 0.46 nA	1.56 × 10^3^, 1.27 × 10^2^, and 1.42 × 10^2^	2.11 × 10^12^, 6.91 × 10^10^, and 6.11 × 10^10^	-	[94]
Ga_2_O_3_/MgO (100)	0.030	3.5 pA	>10^4^	-	0.07/0.06	[59]
Ga_2_O_3_/MgO (100)	0.1	20 pA	>2 × 10^4^	4.3 × 10^12^	-	[58]
Ga_2_O_3_/p-Si (100)	-	~320 nA	~5	-	0.003/0.012	[85]

## Data Availability

No new data were created or analyzed in this study. Data sharing is not applicable to this article.

## Abbreviations

AlGaN	Aluminum gallium nitride
Al	Aluminum
Ar	Argon
APDs	Avalanche photodiodes
BFOM	Baliga figure of merit
I_dark_	Dark current
C	Diamond
E_c_	Energy of conduction band
E_v_	Energy of valence band
EQE	External quantum efficiency
E_u_	Europium
E_f_	Fermi level
FWHM	Full width half maxima
Ga_2_O_3_	Gallium oxide
IQE	Internal quantum efficiency
MIS	Metal–insulator–semiconductor
MSM	Metal–semiconductor–metal
MOCVD	Metal–organic chemical vapour deposition
MBE	Molecular beam epitaxy
PPC	Persistent photoconductivity
PDCR	photo-to-dark current ratio
PDs	Photodetectors
I_photo_	Photo current
PVD	Physical vapour deposition
Pt	Platinum
PLD	Pulse laser deposition technique
RF	Radiofrequency
SBH	Schottky barrier height
Si	Silicon
SIC	Silicon carbide
SNR	Signal-to-noise ratio
SB-UVPDS	Solar-blind UV photodetector
D*	Specific detectivity
Sn	Tin
UV	Ultraviolet
WBGS	Wide bandgap semiconductors
Φ_m_	Work function
ZnMgO	Zinc magnesium oxide
ZnO	Zinc oxide
